# Synergistic halide and phosphate ester electrolytes for overcoming corrosion and interfacial challenges in magnesium batteries

**DOI:** 10.1039/d6sc00095a

**Published:** 2026-02-09

**Authors:** Xuerui Yang, Yuqi Zhou, Junkun Zhou, Xuan Huang, Xin Ao, Guangni Ding, Xiaowei Huang, Naigen Zhou, Guanglei Cui, Yong Yang

**Affiliations:** a School of Physics and Materials Science, Nanchang University Nanchang 330031 China yangxuerui@ncu.edu.cn ngzhou@ncu.edu.cn; b State Key Lab for Physical Chemistry of Solid Surfaces, College of Chemistry and Chemical Engineering, Tan Kah Kee Innovation Laboratory (IKKEM), Xiamen University Xiamen 361005 China yyang@xmu.edu.cn; c Qingdao Industrial Energy Storage Research Institute, Qingdao Institute of Bioenergy and Bioprocess Technology, Chinese Academy of Sciences Qingdao 266101 China cuigl@qibebt.ac.cn; d Ganfeng Lithium Group Co., Ltd, Ganfeng Li Energy Co., Ltd Xinyu 338015 China

## Abstract

The practical development of rechargeable magnesium batteries is fundamentally limited by anode passivation, electrolyte-induced corrosion, and sluggish interfacial Mg^2+^ transport. Herein, we develop a universal electrolyte design strategy that exploits the synergy between halides and phosphate esters to address these long-standing challenges. Typically, the incorporation of SiBr_4_ and tris(trimethylsilyl) phosphate (TMSP) extends the electrochemical stability window of the electrolyte from 2.75 to 3.94 V and reconstructs the solvation environment toward bis(trifluoromethanesulfonyl)imide (TFSI^−^) and TMSP-dominated coordination, significantly lowering the Mg^2+^ desolvation barrier. Preferential reduction of SiBr_4_ and TMSP yields a cross-linked, inorganic-rich interphase comprising Mg_3_(PO_4_)_2_, MgSiO_3_, and MgBr_2_, which enables fast Mg^2+^ transport and effectively suppresses parasitic reactions. Meanwhile, Mg_3_(PO_4_)_2_ and MgSiO_3_ within the interphase serve as robust scaffolds that immobilize soluble MgBr_2_, further reinforcing interfacial stability. Besides, the electron-rich P=O groups in TMSP further stabilize reactive SiBr_3_^+^ intermediates, thereby preventing electrolyte acidification and corrosion. Consequently, Mg‖Mg symmetric cells cycle stably for 1800 h with a low overpotential of 0.14 V. Mg‖Mo cells reach a peak coulombic efficiency of 99.97% at 3.4 V after the activation process. Full cells with a Mo_6_S_8_ cathode deliver a capacity of 80 mAh g^−1^ with only 0.08% fading over 500 cycles, and Mg‖polyaniline–intercalated V_2_O_5_ (PANI–V_2_O_5_) cells achieve 160 mAh g^−1^ at a cut-off voltage of 2.6 V. This synergistic regulation concept is generalizable to other halides and phosphate esters, providing new mechanistic insights and a general framework for designing stable electrolytes for multivalent batteries.

## Abstract 2

A synergistic halide and phosphate ester electrolyte regulates Mg^2+^ solvation and forms a robust inorganic-rich interphase, suppressing corrosion, enhancing Mg^2+^ transport, and providing a general framework for high-voltage multivalent batteries.

## Graphical abstract

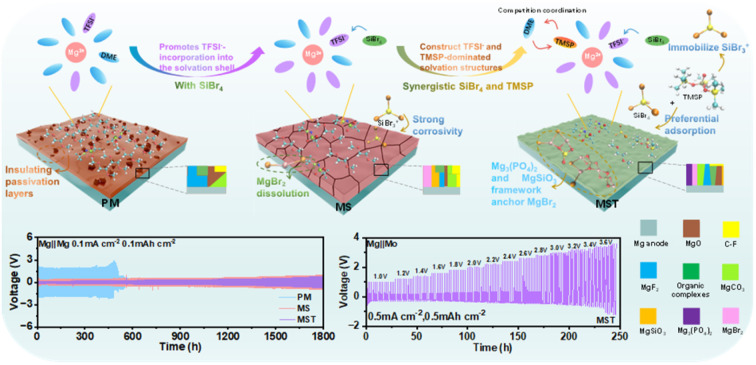

## Introduction

With the growing global demand for efficient and safe energy storage technologies, rechargeable magnesium batteries (RMBs) have attracted considerable attention as promising next-generation high-energy-density systems. Magnesium (Mg) metal offers several intrinsic advantages, including abundant reserves (over 1000 times higher than those of lithium), a high theoretical volumetric capacity of 3833 mAh cm^−3^ (∼1.8 times that of lithium metal, 2061 mAh cm^−3^), and a low redox potential (−2.37 V *vs.* standard hydrogen electrode (SHE)).^1^ More importantly, Mg is inherently resistant to dendrite growth, ensuring a higher safety margin compared with Li, Na, and K metal anodes.^2^ Unfortunately, the practical development of RMBs is severely hampered in commercial electrolytes based on magnesium bis(trifluoromethanesulfonyl)imide (Mg(TFSI)_2_) dissolved in 1,2-dimethoxyethane (DME) electrolyte, where Mg anodes suffer from high overpotentials and rapid performance degradation due to surface passivation and sluggish interfacial electrochemical kinetics.^3^ On the one hand, the high charge-to-radius ratio of Mg^2+^ leads to strong coordination with DME solvent, which significantly increases the desolvation energy barrier.^4^ On the other hand, solvated Mg^2+^ species inevitably induce reductive decomposition of both DME and TFSI^−^ during electrodeposition, forming passivation layers composed of MgO, MgCO_3_, and MgF_2_.^5^ These passivation films possess an extremely low ionic conductivity (10^−9^–10^−8^ S cm^−1^), which drastically impedes Mg^2+^ transport and causes charging overpotentials frequently exceeding 2.2 V.^6^

To address these challenges, various strategies have been proposed, primarily including artificial interphase engineering and electrolyte modification.^7–9^ Artificial interphases are typically fabricated through surface chemical treatments or coating processes to deliberately introduce ion-conductive yet electronically insulating phases at the Mg/electrolyte interface. Common approaches include alloying,^10–12^ halide-based,^13–15^ and polymeric coatings.^16–18^ However, this approach still suffers from inherent limitations, including imbalances between conductive and insulating phases, as well as poor multiscale interfacial compatibility and negligible effect on regulating the solvation structure.^19^

In contrast, electrolyte modification is regarded as a more effective strategy for improving Mg anodes, as it directly regulates solvation structures and enhances interfacial compatibility while maintaining simplicity and cost-efficiency.^20–22^ In particular, strongly coordinating solvents such as amines^4,23^ and phosphate esters^24,25^ have been widely employed to weaken the strong Mg^2+^–ether interactions, thereby lowering the desolvation barrier. However, the interphases derived from their decomposition generally lack long-term stability, resulting in limited overall performance enhancement. Drawing inspiration from classical chloride-based electrolytes [*e.g.*, all-phenyl complex (APC,^26,27^ typically composed of phenylmagnesium chloride (PhMgCl), AlCl_3_, and tetrahydrofuran (THF) as solvents) and magnesium–aluminum/lithium chloride complex (MACC/MLCC,^28,29^ generally consisting of MgCl_2_, AlCl_3_/LiCl, and dimethoxyethane (DME) as solvents)], which enabled highly reversible Mg plating/stripping, recent studies have increasingly focused on incorporating halide additives into conventional Mg(TFSI)_2_–ether systems. This strategy is considered a promising route to reduce the overpotential of Mg plating/stripping. Halides not only form highly ion-conductive interphases *in situ* that facilitate reversible Mg deposition and suppress parasitic reactions, but also reconfigure solvation structures by entering the inner coordination sheath or modulating solvent dipole interactions, thereby weakening Mg^2+^–DME coordination and significantly lowering the interfacial desolvation barrier.^20,30^ For example, Li *et al.*^31^ introduced 3-bromofluorobenzene additives into conventional electrolytes to synergistically regulate solvation structure, solid-electrolyte interphase, and deposition orientation, enabling vertically oriented Mg electrodeposition with enhanced electrochemical performance. Nevertheless, halides suffer from inherent drawbacks such as electrolyte acidification, corrosion of current collectors, and dissolution/migration of halide-derived interphase species, which can further trigger parasitic reactions on the cathode side, especially at high voltage.^32^ Hence, further progress in RMBs relies on rational electrolyte design that simultaneously maximizes beneficial interfacial processes and suppresses adverse side reactions.

Herein, we developed a functional electrolyte based on conventional systems by introducing a synergistic regulation of SiBr_4_ and TMSP, denoted as MST (0.5 M Mg(TFSI)_2_ and 0.35 M SiBr_4_ dissolved in a mixed solvent of DME and TMSP, v/v = 17 : 3). In this system, SiBr_4_ and TMSP cooperatively tailor the anion- and TMSP-dominated solvation sheath, markedly lowering the desolvation barrier on the Mg interface. Meanwhile, their preferential reduction on the Mg surface leads to the *in situ* formation of an inorganic interphase rich in Mg_3_(PO_4_)_2_, MgSiO_3_, and MgBr_2_, thereby accelerating ion transport and effectively suppressing parasitic reactions. In addition, the high oxidative stability of SiBr_4_ and TMSP broadens the electrochemical window of the electrolyte. The electron-rich P=O groups of TMSP stabilize the SiBr_3_^+^ intermediate (upon the elimination of one Br^−^ from SiBr_4_), mitigating further acidification and corrosion. Moreover, MgSiO_3_ and Mg_3_(PO_4_)_2_ in the interphase act as robust scaffolds to immobilize MgBr_2_, reinforcing interfacial stability. Benefiting from these multifaceted synergies, Mg‖Mg symmetric cells exhibit ultrastable cycling with minimal polarization, and Mg‖Mo cells maintain ultrahigh coulombic efficiency even under elevated voltages. Besides, Mg‖Mo_6_S_8_ and Mg‖PANI–V_2_O_5_ full cells deliver high capacity and superior long-term stability. Notably, this synergistic modification strategy demonstrates broad universality and can be readily extended to other halide and phosphate ester-based systems, thereby offering a promising pathway toward practical RMBs.

## Results and discussion

### The effect of solvation structure and interface engineering

**Fig. 1:**
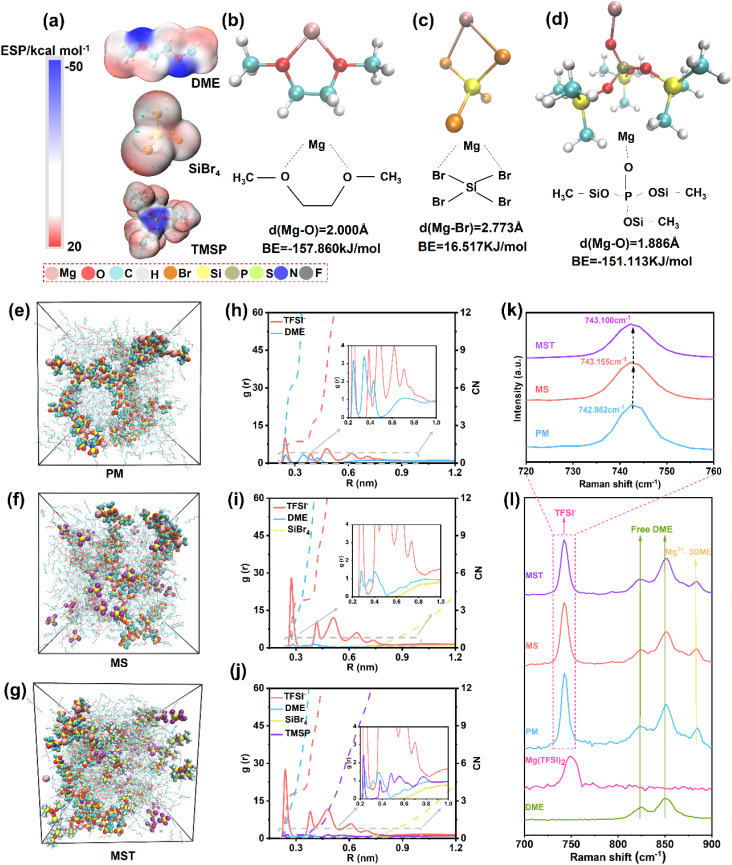
Solvation structure and coordination characteristics of Mg^2+^ in different electrolytes. (a) Electrostatic potential gradient distribution of DME, SiBr_4_, and TMSP. Red and blue regions represent negative and positive charges, respectively. DFT calculation results of coordination structures of (b) Mg^2+^–DME, (c) Mg^2+^–SiBr_4_, and (d) Mg^2+^–TMSP. (e–g) Snapshots and (h–j) radial distribution functions (RDFs) of Mg^2+^ with the TFSI^−^ anion and solvents (DME/SiBr_4_/TMSP) for the (e and h) pristine PM, (f and i) MS, and (g and j) MST electrolytes after MD simulations. (k and l) Raman spectra of the pristine PM, MS, and MST electrolytes in the range of 700–900 cm^−1^.

The interfacial stability and Mg^2+^ transport kinetics are predominantly governed by the intrinsic physicochemical characteristics and solvation behaviors of the electrolyte components. As shown in Fig. 1a, electrostatic potential (ESP) mapping reveals distinct charge distributions among DME, SiBr_4_, and TMSP molecules. In DME, the localized negative charge around the ether oxygen strongly favors coordination with Mg^2+^, forming a five-membered ring-like solvation structure with a binding energy of −157.860 kJ mol^−1^ (Fig. 1b). In contrast, SiBr_4_ exhibits a dominant positive potential around the Si center, and its interaction with Mg^2+^ is thermodynamically prohibited, as evidenced by the positive binding energy of 16.517 kJ mol^−1^ and the longest Mg–Br bond length (2.773 Å, Fig. 1c). The oxygen atom in the P=O group of TMSP carries a pronounced localized negative charge, which enables strong coordination with Mg^2+^. This interaction gives rise to a significantly shorter Mg–O bond length (1.886 Å) and a binding energy of −151.113 kJ mol^−1^ (Fig. 1d), allowing TMSP to effectively compete for Mg^2+^ within the inner solvation shell and thereby reducing the interfacial desolvation barrier. To corroborate this mechanism, molecular dynamics (MD) simulations were performed for three representative electrolyte systems: pristine PM (0.5 M Mg(TFSI)_2_ in DME, Fig. 1e), MS (0.5 M Mg(TFSI)_2_ + 0.35 M SiBr_4_ in DME, Fig. 1f), and MST (Fig. 1g). Radial distribution function (RDF) analysis reveals distinct solvation behaviors across the three electrolytes. In the pristine PM electrolytes, Mg^2+^ is coordinated by an average of 6.26 oxygen atoms from DME and only 1.63 oxygen atoms from TFSI^−^ (Fig. 1h), indicating a DME-dominated primary solvation shell. Upon introducing SiBr_4_ to form the MS electrolyte, the coordination environment shifts to 4.02 (DME) and 3.83 (TFSI^−^) oxygen atoms (Fig. 1i), reflecting a significant replacement of DME by TFSI^−^ in the first solvation shell and the formation of stronger Mg^2+^–TFSI^−^ interactions. Further addition of TMSP in MST electrolytes, the coordination evolves to 3.66 (DME), 3.81 (TFSI^−^), and 0.40 (TMSP) oxygen atoms (Fig. 1j), demonstrating that TMSP competitively binds to Mg^2+^ and further displaces DME from the solvation shell. Together, SiBr_4_ and TMSP synergistically regulate Mg^2+^ solvation, thereby facilitating Mg^2+^ desolvation during interfacial charge transfer.

Complementary Raman spectroscopy was employed to elucidate the ion-association and solvation structures in the Mg(TFSI)_2_-based electrolytes. The ion-pairing states were evaluated based on the S–N stretching vibration of TFSI^−^ located around ∼740 cm^−1^, which is highly sensitive to Mg^2+^–TFSI^−^ interactions.^33,34^ Using the half-peak-width method, the peak positions of this band were determined to be 742.982, 743.155, and 743.100 cm^−1^ for PM, MS, and MST, respectively. The blue shift from PM to MS indicates strengthened Mg^2+^–TFSI^−^ coordination induced by the introduction of SiBr_4_, suggesting enhanced contact ion-pair formation accompanied by weakened Mg^2+^–DME interactions.^35^ Upon further incorporation of TMSP, the slight red shift observed in MST (while remaining higher than that of PM) reflects competitive coordination of Mg^2+^ by the P=O group in TMSP, which partially disrupts Mg^2+^–TFSI^−^ interactions. The 800–900 cm^−1^ region provides additional insight into the DME coordination environment of Mg^2+^. The Raman band at ∼881 cm^−1^ is assigned to coordinated DME species (*e.g.*, Mg^2+^·3DME).^36,37^ The intensity of this band is significantly weaker in MS and MST than in PM, demonstrating that SiBr_4_ and TMSP competitively coordinate with Mg^2+^ and partially replace DME in the primary solvation shell. This competitive solvation facilitates Mg^2+^ desolvation, in good agreement with the MD simulations.

**Fig. 2:**
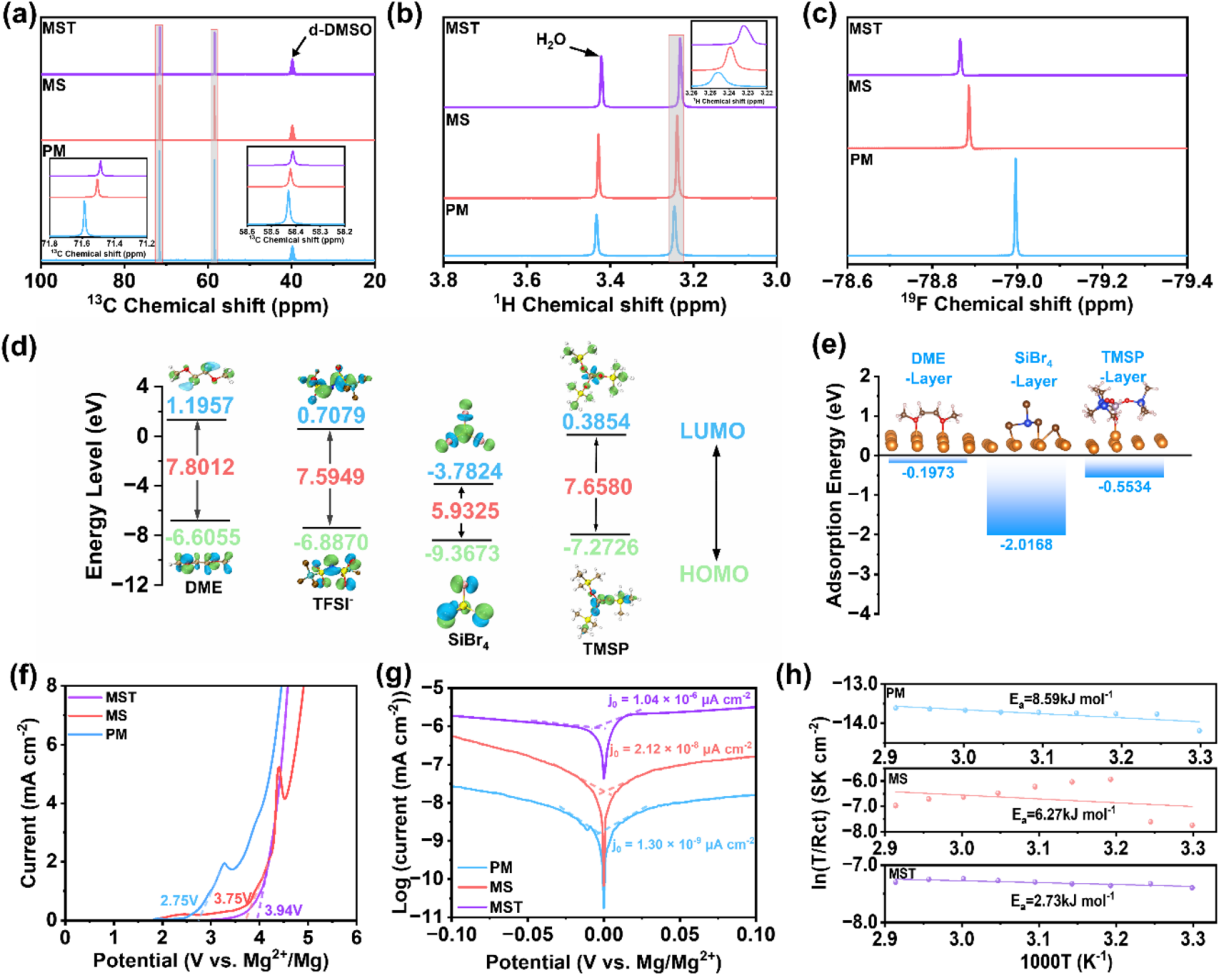
Structural, electronic, and electrochemical analyses of electrolytes. NMR spectra of (a) ^13^C, (b) ^1^H, and (c) ^19^F for the pristine PM, MS, and MST electrolytes. (d) HOMO and LUMO of each component in the electrolyte and its coordination with Mg^2+^. (e) Calculated adsorption energies of DME, SiBr_4_, and TMSP on the Mg (001) crystal plane. (f) LSV curves in the PM, MS, and MST electrolytes at the scan rate of 1 mV s^−1^. (g) Tafel plots obtained from linear sweep polarization measurements for PM, MS, and MST electrolytes. (h) The *E*_a_ values were extracted from the Arrhenius plots of ln(*T*/*R*_ct_) *versus* 1000/*T*.

Nuclear magnetic resonance (NMR) spectroscopy further substantiates these structural insights. In the ^13^C NMR spectra (Fig. 2a), the MS electrolyte exhibits an upfield shift (chemical shift (*δ*) decrease) relative to PM, indicative of enhanced electronic shielding of solvent carbons as Mg^2+^-solvent interactions are weakened.^38^ With further addition of TMSP, the *δ* decreases even more, consistent with competitive coordination between the P=O group and Mg^2+^, which increases electron density around carbon nuclei and reinforces the high-field shift. A similar trend is observed in ^1^H-NMR spectra (Fig. 2b). In contrast, the ^19^F NMR spectra display a downfield shift (*δ* increase) upon addition of SiBr_4_ (Fig. 2c), reflecting stronger integration of electronegative TFSI^−^ anions into the solvation sheath, thereby reducing shielding and exposing fluorine nuclei more directly to the external magnetic field. To probe the intrinsic electrochemical stability of the electrolyte components, frontier orbital energies were evaluated. As shown in Fig. 2d, the relative energy levels of the lowest unoccupied molecular orbital (LUMO) and highest occupied molecular orbital (HOMO) follow the order: SiBr_4_ < TMSP < TFSI^−^ < DME. A lower (more negative) LUMO energy indicates a stronger tendency to accept electrons and thus higher reducibility, whereas a higher (more positive) HOMO energy corresponds to easier electron removal and higher oxidizability.^39,40^ Based on the relative LUMO energies, SiBr_4_ and TMSP are more readily reduced at the Mg anode than DME and TFSI^−^, leading to their preferential decomposition and the formation of a protective solid-electrolyte interphase (SEI), which suppresses continuous electrolyte degradation. In contrast, DME and TFSI^−^, with relatively higher HOMO energies, are more susceptible to oxidation at high potentials. Conversely, the lower HOMO energies of SiBr_4_ and TMSP endow them with greater oxidative stability, thereby contributing to the expanded electrochemical stability window of the electrolyte. The interfacial affinity between solvent molecules and Mg metal was further assessed by adsorption energy calculations (Fig. 2e). The sequence SiBr_4_ (−2.0168 eV) < TMSP (−0.5534 eV) < DME (−0.1973 eV) highlights the preferential adsorption of SiBr_4_ and TMSP on Mg (001), effectively shielding the Mg surface from direct contact with DME and therefore further suppressing parasitic side reactions.

Linear sweep voltammetry (LSV) tests further support the theoretical predictions. The pristine PM electrolyte exhibits a relatively low oxidative decomposition potential of approximately 2.75 V, indicating limited oxidative stability. In contrast, the modified electrolytes show markedly enhanced resistance to oxidation. The onset potential increases to approximately 3.75 V for the MS electrolyte and is further elevated to about 3.94 V for the MST electrolyte (Fig. 2f), demonstrating the effectiveness of the synergistic interaction between TMSP and SiBr_4_ in enhancing oxidative stability. Furthermore, Tafel analysis (Fig. 2g) demonstrates that MST achieves an exchange current density (*j*_0_) of 1.04 × 10^−6^ µA cm^−2^, which is two orders of magnitude higher than that of the MS electrolyte (2.12 × 10^−8^ µA cm^−2^) and nearly three orders of magnitude higher than that of the pristine PM system (1.30 × 10^−9^ µA cm^−2^). The markedly higher *j*_0_ reflects faster Mg plating/stripping enabled by the optimized solvation structure and stabilized interfacial chemistry in the MST electrolyte. Temperature-dependent EIS measurements further elucidate interfacial stability (Fig. S1). In the PM electrolyte, the charge-transfer resistance (*R*_ct_) increases monotonically with temperature, reflecting the aggravation of parasitic reactions at elevated temperatures. The MS electrolyte exhibits an initial decrease in *R*_ct_ from 30 to 40 °C, consistent with accelerated ion transport, but the resistance rises again at higher temperatures as interfacial side reactions prevail. Strikingly, the MST electrolyte displays negligible variation in *R*_ct_ across the full range of 30–70 °C, confirming its ability to suppress electrolyte decomposition and stabilize the electrode/electrolyte interface under thermal stress. To further quantify the interfacial kinetics, the activation energy (*E*_a_) of Mg^2+^ transport was derived from Arrhenius fitting.^41^ The calculated *E*_a_ values for PM, MS, and MST electrolytes are 8.59, 6.27, and 2.73 kJ mol^−1^, respectively (Fig. 2h). The significantly reduced *E*_a_ in MST indicates a much lower Mg^2+^ migration barrier, enabling facile ion transport and rapid charge transfer across the interphase.

### Electrochemical plating/stripping behavior of Mg anodes

**Fig. 3:**
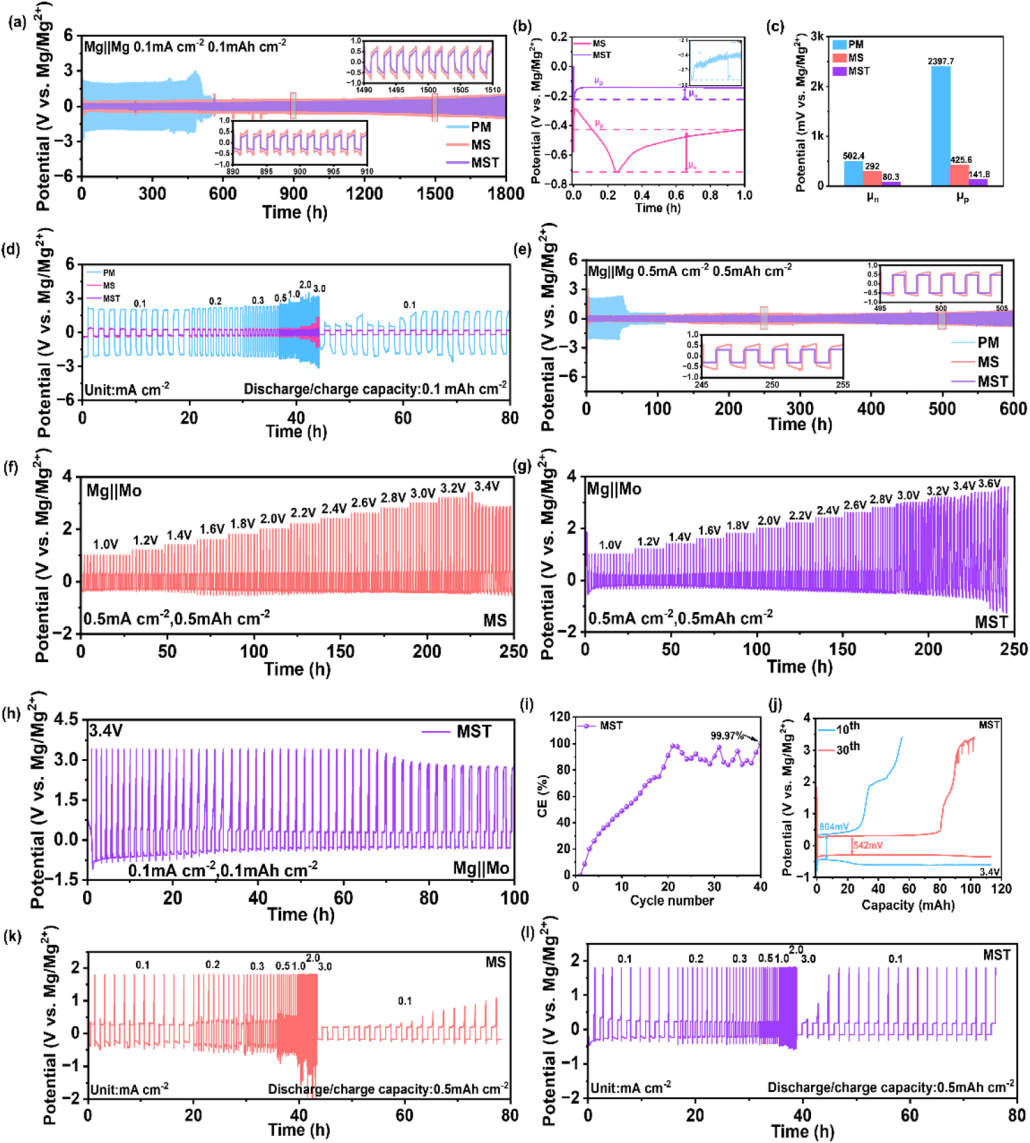
Electrochemical plating/stripping behavior of symmetric Mg‖Mg cells and asymmetric Mg‖Mo cells with different electrolytes. (a–e) Galvanostatic cycling and polarization behavior of symmetric Mg‖Mg cells: (a) long-term cycling at 0.1 mA cm^−2^, 0.1 mAh cm^−2^, with the corresponding (b) initial deposition profile and (c) nucleation (*µ*_n_) and plating (*µ*_p_) overpotentials; (d) polarization responses at a fixed areal capacity of 0.1 mAh cm^−2^ and stepwise current densities from 0.1 to 3.0 mA cm^−2^, followed by extended cycling at 0.1 mA cm^−2^; (e) long-term cycling at 0.5 mA cm^−2^, 0.5 mAh cm^−2^. (f–l) Plating/stripping behavior of the asymmetric Mg‖Mo cells: curves with (f) MS and (g) MST electrolytes at different cut-off voltages ranging from 1.0 V to 3.6 V; (h) curves with MST electrolyte at 0.1 mA cm^−2^ and 3.4 V cut-off and the corresponding (i) Coulomb Efficiency (CE) and (j) voltage–capacity curves at the 10th and 30th cycles; stepwise current density tests from 0.1 to 3.0 mA cm^−2^ at a fixed areal capacity of 0.5 mAh cm^−2^ with (k) MS and (l) MST electrolytes.

To gain deeper insight into the regulatory effects of SiBr_4_ and TMSP on Mg anode behavior, we systematically investigated Mg plating/stripping characteristics at different concentrations of SiBr_4_ and TMSP (Fig. S2). Based on the overall electrochemical performance, the MST electrolyte was selected as the optimized formulation for subsequent electrochemical studies. As shown in Fig. 3a, Mg‖Mg symmetric cells with either the MS or MST electrolyte exhibit ultralong stable operation for over 1800 h at 0.1 mA cm^−2^ and 0.1 mAh cm^−2^. By contrast, cells using the pristine PM electrolyte fail rapidly after only ∼480 h due to short-circuiting. Moreover, the MST-based cells deliver a significantly reduced voltage hysteresis (∼0.43 V), compared with MS (∼0.96 V) and PM (∼4.10 V), highlighting that the formation of a highly conductive SEI is sufficiently robust to substantially enhance the reversibility and durability of Mg plating/stripping. The nucleation overpotential (*µ*_n_) provides further evidence of accelerated charge-transfer kinetics. As shown in Fig. 3b and c, the *µ*_n_ of Mg anodes in MST electrolyte is only 80.3 mV, far lower than in pristine PM (502.4 mV) and MS (282.0 mV). This dramatic reduction indicates that Mg^2+^ ions in MST can readily surmount interfacial energy barriers and nucleate with minimal external polarization. During continued deposition, the plating overpotential (*µ*_p_) decreases progressively. In the PM electrolyte, the deposition plateau stabilizes at an extremely high value of 2.38 V, consistent with severe passivation caused by uncontrolled electrolyte decomposition. By contrast, MST systems exhibit the lowest deposition plateaus of 141.8 mV, respectively, reflecting a more stable interphase and markedly enhanced Mg^2+^ transport across the electrode/electrolyte interface. The polarization response across a wide current-density window (Fig. 3d) further underscores the kinetic advantages of MST. With the pristine PM electrolyte, the overpotentials at 0.1–3.0 mA cm^−2^ increase steeply from ∼2.02 to 3.19 V, revealing severe polarization. MS electrolyte substantially suppresses the overpotentials (0.37–1.35 V), while MST achieves the lowest values (0.24–0.37 V), and uniquely recovers to ∼0.18 V when the current density is reset to 0.1 mA cm^−2^, demonstrating superior reversibility and interfacial stability. At a higher current density of 0.5 mA cm^−2^ with a plating/stripping capacity of 0.5 mAh cm^−2^, cells with MS and MST electrolytes sustain stable cycling for over 600 h, whereas PM fails within ∼60 h due to short-circuiting (Fig. 3e). The corresponding overpotentials decrease drastically from ∼2.10 V in PM to 0.37 V in MS and further to 0.27 V in MST. These results establish that the MST electrolyte enables Mg anodes to show drastically reduced voltage hysteresis, superior rate capability, and ultralong cycling stability.

Subsequently, electrochemical impedance spectroscopy (EIS) was employed to probe the interfacial ion transport properties of Mg‖Mg symmetric cells with different electrolytes. As shown in Fig. S3, the EIS spectra were recorded at the initial state, after 20 cycles, and after 100 cycles, respectively. In the pristine PM electrolyte, the interfacial resistance increases dramatically to ∼175 kΩ after 100 cycles, indicative of severely hindered plating/stripping kinetics caused by the growth of a non-conductive passivation layer on the Mg surface. By contrast, the MS electrolyte shows only a modest increase in resistance, from ∼1.3 to ∼1.8 kΩ over 100 cycles, while the MST electrolyte maintains the lowest resistance and increases only from ∼0.05 to ∼0.5 kΩ, underscoring that the synergistic regulation of SiBr_4_ and TMSP facilitates Mg^2+^ transport and charge transfer at the interface. Moreover, temperature-dependent EIS after 20 cycles, together with *in situ* EIS measurements during electrochemical cycling (Fig. S4), reveals distinct thermal and interfacial behaviors for the MS and MST electrolytes. The MST electrolyte exhibits a continuous decrease in impedance with increasing temperature and stable impedance evolution during cycling, indicating robust Mg^2+^ transport and interfacial kinetics. In contrast, the MS electrolyte exhibits elevated interfacial resistance during cycling and an abrupt impedance increase after 50 °C. This anomalous impedance behavior correlates with a distinct thermal event observed near ∼52 °C in the DSC curve of the MS electrolyte (Fig. S5), as well as with the emergence of additional ^1^H (2.71 ppm) and ^13^C (11.35 ppm) resonances in the NMR spectra after storage at 55 °C (Fig. S6), which are likely associated with thermally induced side reactions between SiBr_4_ and DME.

Fig. 3f and g further evaluates the reversibility of Mg plating/stripping on Mo electrodes at elevated potentials using galvanostatic cycling with progressively increased cut-off voltages. In the PM electrolyte, the Mg plating/stripping exhibits a high overpotential of ∼2.68 V, with coulombic efficiencies of only 1.52% and 4.60% at cut-off voltages of 1.0 and 1.2 V, respectively (Fig. S7). These poor efficiencies highlight severe interfacial side reactions and limited utilization of active Mg, which restricts the electrochemical oxidative window. By contrast, the MS electrolyte markedly reduces the overpotential to ∼0.83 V and delivers coulombic efficiencies above 85% in the range of 1.0–1.6 V (Fig. 3f). However, as the cut-off voltage increases beyond 2.0 V, the coulombic efficiency gradually declines, indicating that the SiBr_4_-derived interphase lacks sufficient oxidative robustness. Strikingly, the MST electrolyte enables Mg‖Mo cells to sustain ultralow hysteresis and nearly ideal coulombic efficiency (∼99%) even at cut-off voltages up to 3.6 V (Fig. 3g), highlighting the formation of a stable, low-impedance interphase capable of suppressing parasitic reactions at high potentials. The long-term reversibility was further examined at a cut-off voltage of 3.4 V. With MST, the Mg‖Mo cell operates stably for ∼70 h (Fig. 3h), during which the coulombic efficiency progressively improves and ultimately approaches ∼99.97% (Fig. 3i). Comparison of the 10th and 30th charge–discharge profiles (Fig. 3j) reveals a distinct charging plateau near 2.0 V, accompanied by a decrease in hysteresis from 0.80 V to 0.54 V, further confirming enhanced reversibility. In contrast, the Mg‖Mo cell with MS electrolyte fails to reach the designated cut-off voltage within 1 h of charging (Fig. S8), underscoring the superior oxidative stability of MST. Rate-dependent galvanostatic cycling further highlights the interfacial robustness of MST. In the PM electrolyte, the Mg‖Mo cell short-circuits at a current density as low as 0.2 mA cm^−2^ (Fig. S9), demonstrating poor interfacial stability. With MS, overpotentials rise progressively from ∼0.40 V at 0.1 mA cm^−2^ to ∼1.89 V at 3.0 mA cm^−2^ (Fig. 3k), accompanied by pronounced voltage fluctuations and rapid polarization growth at ≥1.0 mA cm^−2^. Although the overpotential recovers to ∼0.31 V when the current density is reduced to 0.1 mA cm^−2^, the cell fails to recharge to 1.8 V after 20 cycles, indicating loss of active electrode area. In contrast, the MST electrolyte delivers much lower overpotentials of 0.35–0.62 V across the same current-density range, with rapid recovery to ∼0.25 V upon returning to 0.1 mA cm^−2^. Notably, the MST cell recharges to 1.8 V within only three cycles (Fig. 3l), evidencing superior interfacial reversibility and kinetic stability.

Cyclic voltammetry (CV) measurements further corroborate these findings (Fig. S10). In PM, the first cathodic scan shows a pronounced current, but Mg redox peaks vanish in subsequent cycles, reflecting severe parasitic reactions and irreversibility. In the MS electrolyte, Mg deposition initiates at −0.83 V with a broad and weak cathodic peak, suggesting sluggish nucleation and unstable interfaces. Although the peak intensity increases in later scans, this likely reflects gradual surface reconstruction and continuous interphase thickening. By contrast, MST induces Mg deposition at a much more positive potential of −0.36 V, with sharp, overlapping redox peaks in subsequent cycles, indicative of uniform nucleation and a compact, low-resistance SEI that stabilizes cycling. Surface characterization provides direct evidence for these interfacial improvements. Scanning electron microscopy (SEM) and energy dispersive spectrometer (EDS) analyses reveal that Mg deposited in MST forms a dense and uniform layer on the Mo current collector, while deposition in MS yields irregular and granular morphologies (Fig. S11).

### Surface morphologies and interphase evolution

**Fig. 4:**
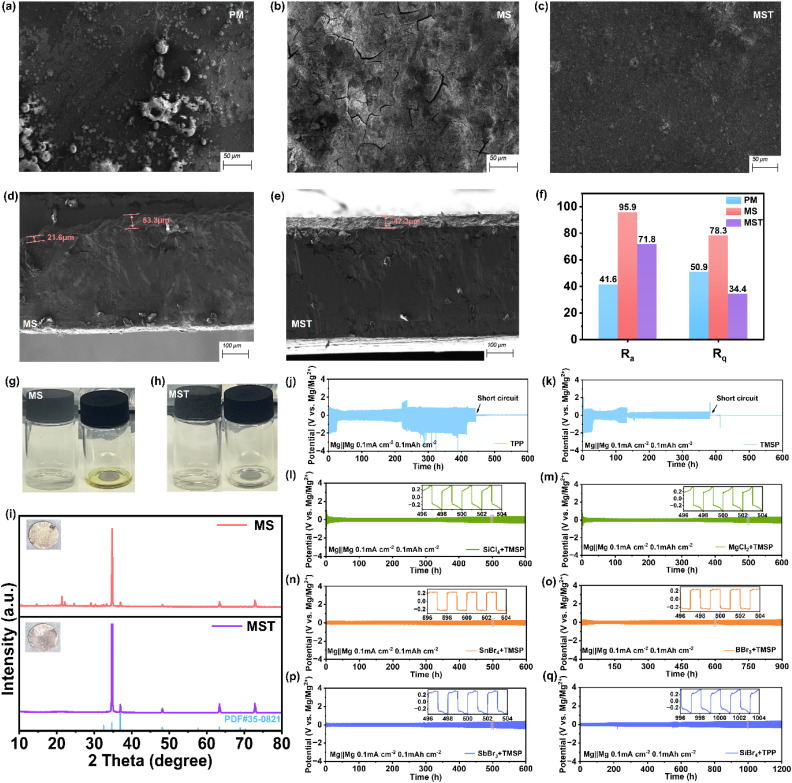
Surface morphologies and structural evolution of Mg anodes in different electrolytes. SEM images of the magnesium anode after 100 cycles, showing surface morphologies in the (a) pristine PM, (b) MS, and (c) MST electrolytes, as well as cross-sectional views in the (d) MS and (e) MST electrolytes. (f) The corresponding statistical analysis of surface roughness parameters *R*_a_ (arithmetic mean deviation of the profile) and *R*_q_ (root mean square roughness) at different electrolytes. Photographs of the Mg foil after immersion in the (g) MS and (h) MST electrolytes for 30 min, and (i) XRD patterns of the immersed Mg foils. (j–q) Cyclic performance of Mg‖Mg symmetric cells assembled with halide and phosphate ester co-modified electrolytes, as well as with single-phosphate ester electrolytes (triphenyl phosphate (TPP) or TMSP).

A scanning electron microscope (SEM) was used to investigate the morphological evolution of Mg anodes before and after cycling. Compared with the relatively smooth surface of pristine Mg (Fig. S12), the anode cycled for 100 cycles in the pristine PM electrolyte exhibits a rough surface with numerous particulate protrusions (Fig. 4a). Upon further cycling to short-circuiting failure, the Mg surface develops irregular pits and pronounced groove-like cracks (Fig. S13), indicative of highly uneven deposition and the continuous accumulation of interfacial by-products. Meanwhile, EDS further confirms the enrichment of C, O, and F elements, evidencing decomposition of the DME solvent and Mg(TFSI)_2_ salt as the primary source of parasitic layers. In the MS electrolyte, Mg deposits appear relatively uniform and compact, yet the interphase remains mechanically unstable. Pronounced surface cracks are observed after 300 cycles (Fig. 4b), accompanied by significant aggregation of Si and Br elements (Fig. S14). Cross-sectional SEM reveals step-like heterogeneous structures (Fig. 4d) and microcracks penetrating the interphase (Fig. S15), suggesting that the SiBr_4_-derived interphase is prone to degradation, likely through corrosion by acidic by-products and the dissolution of MgBr_2_.^42^ In contrast, the Mg anode cycled in MST electrolyte retains a smooth surface (Fig. 4c) with a compact granular morphology and homogeneous elemental distribution even after 300 cycles (Fig. S16). Cross-sectional analysis confirms the formation of a dense and intact interphase without destructive cracks (Fig. 4e), underscoring the stabilizing role of MST. Atomic force microscopy (AFM) provides direct and quantitative evidence of these interfacial differences. After cycling in PM, Mg electrodes exhibit a severely roughened surface, consistent with a fragile and loose interphase incapable of regulating uniform deposition (Fig. S17). The MS electrolyte partially mitigates morphological instability but still displays pronounced height fluctuations, indicative of an interphase vulnerable to acidic degradation (Fig. S18). Strikingly, the MST electrolyte yields a compact and uniform surface with significantly reduced roughness. Statistical analysis of arithmetical mean deviation (*R*_a_) and root-mean-square roughness (*R*_q_) (Fig. 4f) further highlights that MST achieves the lowest *R*_q_ despite a moderately higher *R*_a_, reflecting homogeneous morphology with suppressed extreme deviations. Notably, the anomalously low values in PM likely stem from interphase delamination during disassembly rather than genuine surface stability (Fig. S19). To directly assess the corrosive nature of MS and the corrosion-inhibiting ability of MST, Mg foils were immersed in the two electrolytes for 0.5 h. Pronounced discoloration occurs in MS (Fig. 4g), while the solution with MST remains clear and transparent (Fig. 4h). X-ray diffraction (XRD) patterns support these findings. Mg foils in MS exhibit suppressed Mg signals and emergent impurity peaks, whereas those in MST retain sharp Mg reflections without notable impurities (Fig. 4i). Similar trends are observed across other halide and phosphate ester-based systems (Fig. S20). Halide-only electrolytes are inherently corrosive, whereas the incorporation of phosphate esters suppresses corrosion and stabilizes Mg. Indeed, Mg‖Mg cells with mixed halide and phosphate ester electrolytes exhibit both low overpotentials (<0.2 V) and long-term cycling stability, outperforming phosphate ester-only counterparts that provide little improvement (Fig. 4j–q). Theoretical calculations further elucidate the corrosion-inhibition mechanism (Fig. S21). Halide-derived reactive Lewis-acidic species (*e.g.*, SiBr_3_^+^) generated during dehalogenation readily attack the Mg surface. Phosphate esters mitigate this effect by coordinating with these species, thereby neutralizing their reactivity and suppressing interfacial degradation.

**Fig. 5:**
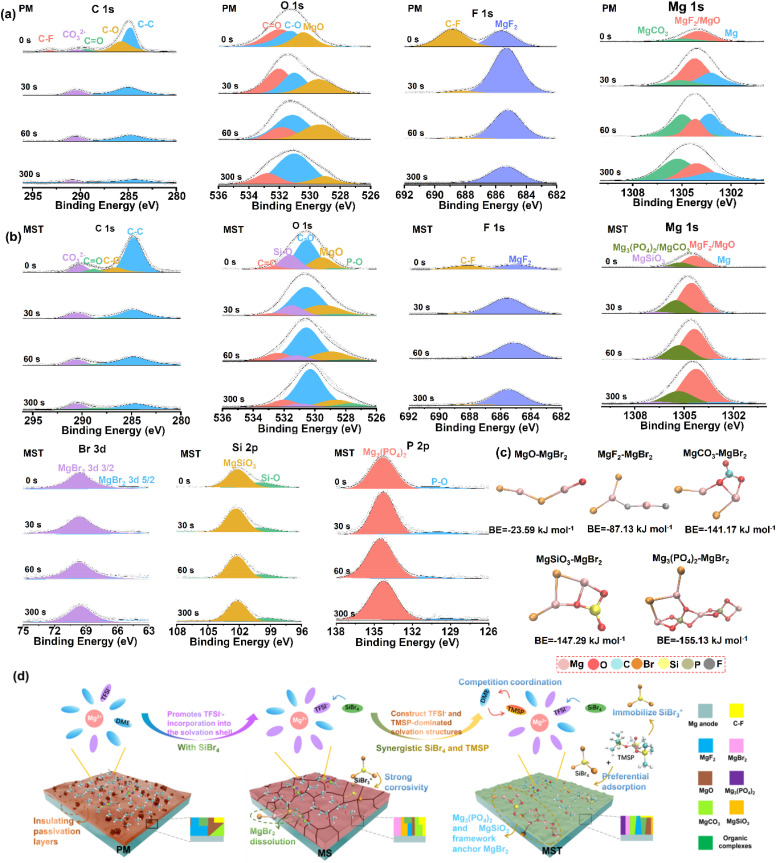
Interphase composition and phase formation processes on Mg anodes in different electrolytes. XPS spectra (C 1s, O 1s, F 1s, Mg 1s, Br 3d, Si 2p, and P 2p) of the magnesium anode cycled for 100 cycles in the (a) PM and (b) MST electrolytes at different Ar^+^ sputtering times. (c) Calculated binding energies between MgBr_2_ and various interphase components (MgO, MgF_2_, MgCO_3_, MgSiO_3_, Mg_3_(PO_4_)_2_). (d) Schematic representation of solvation structures in different electrolytes and electrolyte-dependent interfacial phase evolution.

X-ray photoelectron spectroscopy (XPS) was conducted to elucidate the interfacial chemistry of Mg anodes cycled in different electrolytes (Fig. 5a, b and S22). In the C 1s spectra, the PM electrolyte exhibits prominent peaks at ∼284.8 eV (C–C), ∼286.3 eV (C–O), and ∼287.4 eV (C=O),^43^ which are attributed to solvent-derived organic species. The relative intensities of these peaks follow the order PM > MS > MST (Fig. S23), underscoring that MST most effectively suppresses solvent decomposition. The O 1s spectra reveal components of MgO (∼530.0 eV), C=O (∼531.5 eV), and C–O (∼533.0 eV).^44^ The co-existence of strong C=O and C–O signals, together with the CO_3_^2−^ peak in the C 1s spectra (∼289.0 eV),^45^ suggests the formation of carbonate-rich species (*e.g.*, MgCO_3_). The Mg 1s spectra further support this result, with a peak at ∼1305.4 eV characteristic of MgCO_3_, mainly arising from the deep decomposition of the DME solvent.^44,46^ The F 1s spectra show two characteristic peaks at ∼687.5 eV for C–F^46^ and ∼685.0 eV for MgF_2_,^47,48^ also observed in Mg 1s spectra at ∼1304.8 eV, which are typical products of TFSI^−^ decomposition. These species (MgCO_3_, C–F, and MgF_2_) are most abundant in PM, reduced in MS, and minimized in MST (Fig. S23), providing direct evidence that MST suppresses electrolyte decomposition. Furthermore, depth profiling by Ar^+^ sputtering reveals a compositional gradient in which inorganic species (MgO, MgCO_3_, MgF_2_) are enriched in the inner interphase, while organic moieties are concentrated near the surface. Notably, metallic Mg signals (∼1303.0 eV) detected in PM indicate local exposure of Mg metal caused by an unstable and non-uniform interphase. In contrast, such signals are negligible in MST, reflecting the formation of a compact and continuous interphase that maintains interfacial integrity during cycling. Distinct differences emerge in the MS and MST systems. In MS, Br 3d and Si 2p peaks correspond to MgBr_2_,^49^ and MgSiO_3_/Si–O^50^ species, with intensities increasing upon sputtering, suggesting their enrichment in the inner SEI. In contrast, the MST electrolyte produces weaker MgBr_2_ and MgSiO_3_ signals that remain nearly constant with depth. Similarly, the Mg_3_(PO_4_)_2_ features in the P 2p spectra^51,52^ exhibit negligible depth dependence, further indicating that the SEI derived by MST is compact and uniformly inorganic-rich, thereby more effectively suppressing parasitic reactions. To clarify the stability contributions, binding energy calculations (Fig. 5c) demonstrate that the Mg_3_(PO_4_)_2_ and MgSiO_3_ frameworks in the SEI exhibit strong anchoring effects on soluble MgBr_2_, thereby reinforcing interphase integrity. These mechanistic insights are summarized schematically in Fig. 5d. In PM, uncontrolled solvent decomposition results in a thick, heterogeneous SEI enriched in MgO, MgCO_3_, MgF_2_, and organic compounds. With SiBr_4_ (MS electrolyte), the solvation environment shifts to anion-dominated shells, forming an SEI primarily composed of MgF_2_, MgSiO_3_, and MgBr_2_. However, the stability of this interphase may be compromised by the dissolution of MgBr_2_ and the highly reactive intermediates of SiBr_3_^+^. By contrast, in MST, the synergistic interplay of SiBr_4_ and TMSP directs the formation of anion- and TMSP-coordinated solvation structures that lower the desolvation barrier, while concurrently yielding a high ionic conductivity inorganic-rich SEI (mainly MgSiO_3_, MgBr_2_, and Mg_3_(PO_4_)_2_). Importantly, MgSiO_3_ and Mg_3_(PO_4_)_2_ within the SEI can immobilize otherwise soluble MgBr_2_, while phosphate groups from TMSP coordinate with acidified SiBr_3_^+^ species, thereby suppressing halide-induced corrosion and reinforcing interphase robustness.

### Performance verification of full cells

**Fig. 6:**
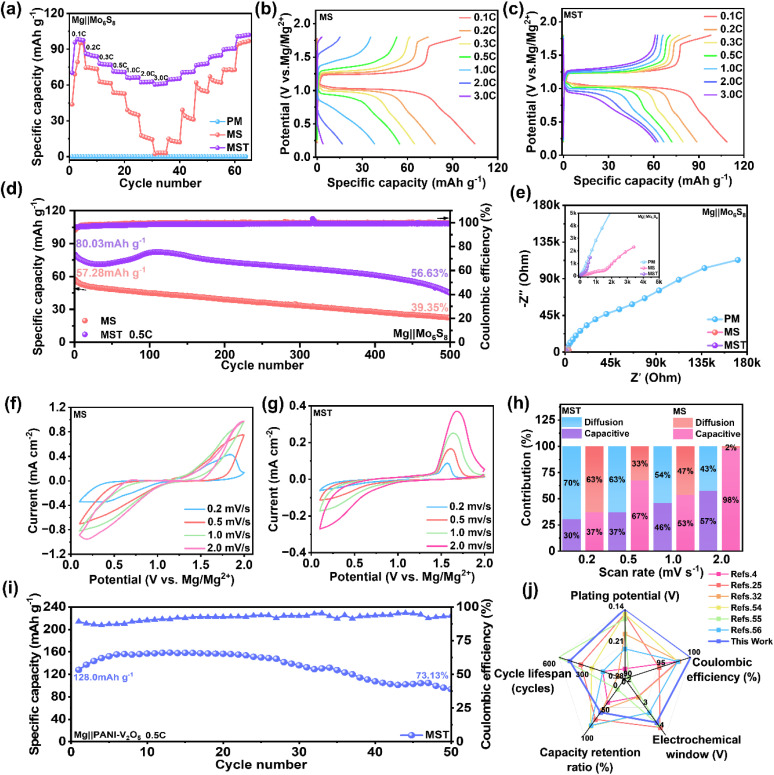
Electrochemical performance of Mg‖Mo_6_S_8_ and Mg‖PANI–V_2_O_5_ full cells with different electrolytes. (a–h) The electrochemical performance of Mg‖Mo_6_S_8_ full cells: (a) Rate performance with PM, MS, and MST electrolytes, respectively; galvanostatic charge–discharge profiles with (b) MS and (c) MST electrolytes at various current densities (0.1–3.0 C); (d) cycling performance at 0.5 C; (e) EIS spectra before cycling with PM, MS, and MST electrolytes; CV curves at scan rates of 0.2–2.0 mV s^−1^ with MS (f) and MST (g) electrolytes, and the (h) fitted capacitance and diffusion contributions of Mo_6_S_8_. (i) Cycling stability of Mg‖PANI–V_2_O_5_ cells with the MST electrolyte at 0.5 C. (j) Radar plot comparing the key electrochemical performance metrics of the MST electrolyte with representative electrolytes reported in the literature.

To further assess the practical applicability of the MST electrolyte, Mg‖cathode full-cell configurations were systematically investigated. The Chevrel-phase Mo_6_S_8_ cathode was synthesized as reported previously,^53^ and XRD analysis confirms both the intermediates and the final Chevrel-phase structure (Fig. S24). Fig. 6a–c first compares the rate performance of Mg‖Mo_6_S_8_ full cells in different electrolyte systems. In the pristine PM electrolyte, the deliverable capacity is nearly negligible at all rates, reflecting severe interfacial instability and sluggish Mg^2+^ transport kinetics that hinder effective Mg^2+^ intercalation/extraction (Fig. 6a). Upon modification with SiBr_4_ (MS electrolyte), the cells exhibit appreciable capacity at 0.1 C (1 C = 100 mA g^−1^), but suffer a sharp decline with increasing current density, accompanied by incomplete capacity recovery upon returning to 0.1 C, indicative of pronounced polarization and poor reversibility. By contrast, the MST electrolyte maintains significantly higher capacities across a broad range of rates, with nearly full capacity recovery at 0.1 C, highlighting its excellent rate capability and electrochemical reversibility. These trends are corroborated by the charge–discharge profiles (Fig. 6b and c) and GITT measurements (Fig. S25). While the MS system shows rapidly widening polarization at elevated rates (Fig. 6b), the MST system preserves well-defined voltage plateaus with minimal hysteresis (Fig. 6c). GITT further confirms that MST delivers higher capacities and stable voltage profiles across multiple current steps (Fig. S25), and the calculated Mg^2+^ diffusion coefficients (*D*_Mg_^2+^) demonstrate consistently faster ion transport, especially in the medium-to-high voltage region, underscoring the superior transport kinetics enabled by the synergistic SiBr_4_–TMSP design.

Long-term cycling tests further validate these improvements. As shown in Fig. 6d, Mg‖Mo_6_S_8_ full cells at 0.5 C retain 45 mAh g^−1^ after 500 cycles (57% retention) in MST electrolyte, compared with only 23 mAh g^−1^ (39% retention) in MS. Importantly, MST cells sustain coulombic efficiencies above 99% throughout, reflecting a stable SEI that effectively suppresses parasitic reactions. EIS analysis (Fig. 6e) consistently reveals that MST delivers much lower charge-transfer resistance than both MS and PM, supporting its superior interfacial kinetics. CV further elucidates the kinetic origin of these enhancements (Fig. S26). In PM, redox peaks are essentially absent, confirming extremely poor electrochemical activity. The MS system exhibits broad and poorly defined peaks with large polarization, consistent with unstable interfacial evolution. By contrast, the MST system displays distinct and stable oxidation peaks (∼1.5–2.0 V) from the initial cycle, with intensities progressively increasing and minimal shifts, indicative of stable interfaces and sustained reversibility. Scan rate-dependent CV (0.2–2.0 mV s^−1^) further distinguishes the two systems (Fig. 6f and g). Although both exhibit increasing currents with scan rate, MST preserves sharp and well-defined peaks even in the high-potential region, indicative of accelerated redox kinetics and superior interfacial compatibility. Quantitative analysis (Fig. 6h, based on Fig. S27) reveals that MST is predominantly diffusion-controlled (70% at 0.2 mV s^−1^ and 43% at 2.0 mV s^−1^), whereas MS exhibits a largely pseudocapacitive response. Such diffusion-dominated kinetics highlight the battery-type storage nature of MST, which is intrinsically advantageous for achieving higher capacity.

Given these favorable characteristics, the applicability of MST was further extended to high-voltage energy storage. Mg‖polyaniline–intercalated V_2_O_5_ (PANI–V_2_O_5_) cells were evaluated, where polyaniline intercalation was employed to enhance Mg^2+^ diffusion of V_2_O_5_. XRD and SEM confirm the structural and morphological integrity of PANI–V_2_O_5_ (Fig. S28 and S29). With MST electrolyte, these cells deliver 94 mAh g^−1^ after 50 cycles with 73% retention and nearly 100% coulombic efficiency (Fig. 6i). Voltage-capacity profiles (Fig. S30) display highly stable charge/discharge plateaus with minimal hysteresis, while CV (Fig. S31) and EIS (Fig. S32) consistently demonstrate superior interfacial stability and rapid kinetics of the MST system. Finally, a systematic comparison with previously reported Mg(TFSI)_2_/magnesium trifluoromethanesulfonate (Mg(OTf)_2_)-based electrolyte systems (Table S1 and Fig. 6j)^4,25,32,54–56^ clearly demonstrates the superior overall performance of the present formulation. More importantly, the mixed halide and phosphate ester synergistic regulation strategy can be readily extended to other magnesium salt-based systems. As illustrated in Fig. S33, Mg‖Mg cells with the formulation of 0.3 M magnesium tetrakis(hexafluoroisopropoxy)borate (Mg(B(HFIP)_4_)_2_) and 0.35 M SiBr_4_ in a DME : TMSP (17 : 3, v/v) mixture exhibit stable cycling for over 1800 hours with an overpotential below 0.25 V, further validating the universality and effectiveness of this electrolyte design principle.

## Conclusions

In summary, this work proposes an electrolyte regulation strategy for magnesium-ion batteries based on the synergistic effect of SiBr_4_ and TMSP in conventional electrolytes. For one thing, the synergistic introduction of SiBr_4_ and TMSP in MST electrolyte promotes TFSI^−^ and TMSP-dominated solvation structures, which effectively reduce the interfacial desolvation barrier. Additionally, both SiBr_4_ and TMSP are preferentially reduced on the Mg anode surface to form a robust and uniform inorganic-rich interphase composed of Mg_3_(PO_4_)_2_, MgSiO_3_, and MgBr_2_, which suppresses parasitic reactions and enhances Mg^2+^ transport kinetics. Within the interphase, Mg_3_(PO_4_)_2_ and MgSiO_3_ act as a structural framework that anchors MgBr_2_ species, thereby further reinforcing interfacial stability. Meanwhile, the electron-rich P=O groups in TMSP stabilize Br^−^-eliminated SiBr_3_^+^ intermediates, mitigating electrolyte acidification and corrosion. Moreover, the excellent oxidative stability of SiBr_4_ and TMSP also contributes to an extended electrochemical window of 3.94 V. Owing to these advantages, the MST electrolyte exhibits significantly improved electrochemical performance. Mg‖Mg symmetric cells demonstrate stable cycling over 1800 hours with a reduced nucleation overpotential of 80.3 mV. Mg‖Mo cells achieve reversible plating/stripping within an extended voltage window up to 3.6 V with coulombic efficiency as high as 99% and a current density limit of 3.0 mA cm^−2^. In full-cell configurations, Mg‖Mo_6_S_8_ full cells deliver an initial capacity of 80 mAh g^−1^ at 0.5 C with an ultralow fading rate of 0.08% per cycle over 500 cycles and retain 63 mAh g^−1^ even at 3 C. Moreover, the Mg‖PANI–V_2_O_5_ cells with the MST system also demonstrate good compatibility with 73% retention after 50 cycles and nearly 100% coulombic efficiency. Importantly, this strategy can also be extended to other halides and phosphate esters, offering a general framework and promising direction of electrolyte design for high-performance magnesium batteries.

## Author contributions

Xuerui Yang: conceptualization, methodology, investigation, data curation, funding acquisition, writing – review & editing. Yuqi Zhou: formal analysis, investigation, data curation, writing – original draft. Junkun Zhou: methodology, investigation, and data curation. Xuan Huang: formal analysis, and data curation. Xin Ao: writing – review & editing. Guangni Ding: formal analysis. Xiaowei Huang: data curation. Naigen Zhou: funding acquisition. Guanglei Cui: data curation, and writing – review & editing. Yong Yang: data curation, funding acquisition, and writing – review & editing.

## Conflicts of interest

The authors declare no competing interests.

## Supplementary Material

SC-OLF-D6SC00095A-s001

## Data Availability

The data supporting this article have been included as part of the supplementary information (SI). Supplementary information is available. See DOI: https://doi.org/10.1039/d6sc00095a.
