# Supplementary material for: Synergistic halide and phosphate ester electrolytes for overcoming corrosion and interfacial challenges in magnesium batteries
Source: Chem Sci. 2026 Feb 9;17(14):7102–14. doi: 10.1039/d6sc00095a (PMC12908025; doi:10.1039/d6sc00095a)
Supplement: SC-017-D6SC00095A-s001 [file SC-017-D6SC00095A-s001.pdf]

Supplementary Information (SI) for Chemical Science.
This journal is © The Royal Society of Chemistry 2026

Supporting Information for

Synergistic Halide and Phosphate Ester Electrolytes for Overcoming Corrosion and Interfacial Challenges in Magnesium Batteries

Xuerui Yang [a, d, *, #], Yuqi Zhou [a, #], Junkun Zhou [a], Xuan Huang [a], Xin Ao [a], Guangni Ding [a], Xiaowei Huang [d], Naigen Zhou [a, *], Guanglei Cui [c, *], and Yong Yang [b, *]

[a] *School of Physics and Materials Science, Nanchang University, Nanchang, 330031, China*

[b] *State Key Lab for Physical Chemistry of Solid Surfaces, College of Chemistry and Chemical Engineering & Tan Kah Kee Innovation Laboratory (IKKEM), Xiamen University, Xiamen, 361005, China*

[c] *Qingdao Industrial Energy Storage Research Institute, Qingdao Institute of Bioenergy and Bioprocess Technology, Chinese Academy of Sciences, Qingdao, 266101, China*

[d] *Ganfeng Lithium Group Co., Ltd., & Ganfeng Li Energy Co., Ltd. Xinyu, 338015, China*

[*] *Corresponding author; E-mail address: yangxuerui@ncu.edu.cn, ngzhou@ncu.edu.cn, cuigl@qibebt.ac.cn, yyang@xmu.edu.cn*

[#] *Authors contributed equally*

Experimental Section

Electrolyte preparation

A 0.5 M $Mg(TFSI)_2$ (magnesium bis(trifluoromethylsulfonyl)imide) solution in DME (1,2-dimethoxyethane), denoted as PM electrolyte, was purchased from Suzhou Duoduo Chemical Technology Co., LTD. Mixed-solvent electrolytes were then prepared to systematically investigate the combined effects of $SiBr_4$ concentration and TMSP (tris(trimethylsilyl) phosphate) content. Specifically, 0.5 M $Mg(TFSI)_2$ electrolytes containing varying concentrations of $SiBr_4$ (99.99% metals basis, Adamas; 0.2 M, 0.35 M, and 0.5 M) in DME:TMSP (Suzhou Qitian New Material Co., Ltd.) mixed solvents with volume ratios of 16:4, 17:3, and 18:2 were prepared. All electrolytes were prepared by stirring the corresponding components at 25 °C in an argon-filled glovebox until clear solutions were obtained. Molecular sieves (4 Å) were subsequently added to remove residual moisture, and the electrolytes were stored in the glovebox before use. All preparation procedures were carried out under an argon atmosphere with H_2O and O_2 concentrations maintained below 0.1 ppm. Based on the cycling performance of Mg‖Mg symmetric cells shown in Fig. S2, the electrolyte composed of 0.5 M $Mg(TFSI)_2$, 0.35 M $SiBr_4$, and a DME:TMSP volume ratio of 17:3 exhibited the most stable and prolonged cycling behavior. This optimized formulation is denoted as the MST electrolyte and was therefore selected for subsequent electrochemical studies. For comparison and extended investigations, additional electrolytes were also prepared under identical conditions, including 0.5 M $Mg(TFSI)_2$ + 0.35 M $SiBr_4$ in DME (denoted as MS), 0.5 M $Mg(TFSI)_2$ in DME:TMSP (17:3, v/v), as well as electrolytes containing alternative halide additives ($SiCl_4$, BBr_3, $SnBr_4$, and $MgCl_2$) or phosphorus-containing solvents (TPP (triphenyl phosphate) and TMSP) at identical concentrations and solvent volume ratios.

Electrode preparation

Mg anode preparation. Mg foils (99.95 %, 0.3 mm thick) were purchased from XinXiang YouYueXin Metal Materials Technology Co., LTD. Then the Mg foils were cut into disks with a diameter of 15 mm and polished with a 2500-grit sandpaper in an argon-filled glovebox (with H_2O < 0.1 ppm and O_2 < 0.1 ppm) to remove the oxide layer.

Mo_6S_8 cathode preparation. The Chevrel Mo_6S_8 was synthesized using a modified procedure from the previous work[1]. Briefly, 0.8 g of CuS, 1.6 g of MoS_2, 1.2 g of Mo powder, and 16 g of KCl were mixed and ball-milled at 350 rpm for 3 h. The ground powder was then transferred to a crucible for sintering. Under a sealed argon atmosphere, the sample was sintered in a tubular furnace at 300 °C for 1 h, then, the

sample was heated up to 1100 °C for another 24 h. After cooling to room temperature, the products were washed with deionized water, and $Cu_2Mo_6S_8$ could be obtained. As prepared, $Cu_2Mo_6S_8$ was then immersed in a 6 M HCl solution and stirred for 72 h at 40 °C. The solution was centrifuged, and the Mo_6S_8 precipitates were washed with deionized water and dried under vacuum at 80 °C. The Mo_6S_8 cathodes were prepared by spreading the mixed slurry composed of 70wt. % Mo_6S_8 powder, 20wt. % super P, and 10wt. % polyvinylidene fluoride (PVDF) in the NMP solvent to Mo foils and dried at 80 °C. The mass loading of the active material is ~2.0 mg cm^{-2}.

PANI-intercalated V_2O_5 cathode preparation. The PANI-intercalated V_2O_5 was prepared by hydrothermal reaction[2]. Typically, 0.18 g of commercial V_2O_5 powder was dispersed in 30 mL of deionized water and vigorously stirred at room temperature. Then, 60 μL aniline was added to the precursor solution and continuously stirred. Next, concentrated HCl was dropped into the above mixture to adjust its pH to 3. After stirring at room temperature for 30 minutes, the mixture was transferred to a 50 mL Teflon lined stainless steel autoclave. The autoclave is heated at 120 °C for 24 h and then cooled to room temperature naturally. The product was washed with deionized water and anhydrous ethanol for 3 times. Finally, the obtained samples were dried under vacuum at 60 °C for 10 h before using. The PANI-intercalated V_2O_5 cathode paste was prepared by mixing 70wt. % V_2O_5, 20wt. % CNT and 10wt. % PVDF evenly. Then the cathode paste was cast on titanium foils with a diameter of 12 mm. The mass loading of the active material is ~2.0 mg cm^{-2}.

Battery assembly and the electrochemical measurements

Mg‖Mg symmetric cells, Mg‖Mo asymmetric cells, and Mg‖Mo_6S_8/PANI-V_2O_5 full cells were assembled by using CR2025-type coin cells with glass fiber (Whatman GF/D) as the separator and 200 μL predetermined electrolytes. All cell assemblies were conducted in an Ar-filled glove box (H_2O < 0.1 ppm and O_2 < 0.1 ppm). The battery performance was evaluated using a multi-channel battery test system (Shenzhen Xinwei Electronics Co., LTD). Linear sweep voltammetry (LSV) was used to study the oxidation stability of different substrates in the PM, MS and MST electrolytes, which was conducted at a scan rate of 1 mV s^{-1} in coin cells with molybdenum (Mo) foil as the working electrode and polished Mg foil as the anode. Cyclic voltammetry (CV) measurement based on asymmetric cells (from -1 V to 2 V vs. Mg/Mg^{2+}), Mg‖Mo_6S_8 full cells (from 0.2 V to 1.8 V vs. Mg/Mg^{2+}), and Mg‖PANI-V_2O_5 full cells (from 0.1 V to 2.6 V vs. Mg/Mg^{2+}) was conducted using Biologic work station at a scan rate of 0.5 mV s^{-1}. Tafel curves based on symmetric cells were obtained from -100 mV to 100 mV at a scan rate of 0.1 mV s^{-1}. The corresponding exchange current density was calculated by the Butler-Volmer Equation. Electrochemical impedance

spectroscopy (EIS) characterizations of cells were carried out using an amplitude of 10 mV with frequencies between 100 MHz and 0.1 Hz by the Solartron Frequency Analyzer. To further quantify the interfacial kinetics, the activation energy (E_a) was derived using the Arrhenius relation:

$$1/R_{ct} = A\exp(-E_a/RT)$$

where A is the pre-exponential factor, R is the gas constant, and T is the temperature.

Material characterizations

The morphology of the Mg anode was examined using scanning electron microscopy (SEM) (ZEISS/Sigma 560, ZEISS), coupled with an energy dispersive X-ray spectrometer (EDS) to determine elemental composition. X-ray diffraction (XRD) patterns were measured on an X-ray diffractometer (D8 Advance, Bruker) with Cu-K_α radiation under 40 kV and 40 mA. X-ray photoelectron spectroscopy (XPS) (Thermo SCIENTIFIC Nexsa) experiments were performed in a Theta probe using monochromatic Al K_α X-rays at hν = 1486.6 eV. Raman spectra of PM, MS, and MST electrolytes were collected with DXR3xi Raman Microscope (Thermo Fisher Scientific) using the 532 nm He-Ne laser source. PM, MS, and MST electrolytes were used for nuclear magnetic resonance (NMR) spectroscopy measurement by a Bruker Avance NEO 400 spectrometer. 3 mg of the sample was used for Mettler-Toledo differential scanning calorimetry (DSC), and the measurement was conducted under the protection of a nitrogen atmosphere with a heating rate of 1 °C·min^{-1} from 25 °C to 70 °C. All the electrodes were disassembled from the coin cells in an Ar-filled glove box (H_2O < 0.1 ppm and O_2 < 0.1 ppm). The electrodes were washed with 1,2-dimethoxyethane (DME) solvent and dried at room temperature for further characterization.

Theoretical calculations

Classical Molecular Dynamics (CMD) Simulation Methods. All the classical molecular dynamics simulations in this work are performed by the Gromacs 2020.3 package[3]. The boxes with the size of 10×10×10 nm^3 containing 962 DME, 50 Mg^{2+}, 100 $TFSI^-$, 962 DME, 50Mg^{2+}, 100 $TFSI^-$, 37$SiBr_4$ and 962 DME, 50 Mg^{2+}, 100 $TFSI^-$, 37$SiBr_4$, 46 TMSP respectively, were built by Packmol software[4]. General amber force field (GAFF) parameters were generated by Sobtop[5]. The conjugated gradient method was used for energy minimization. The equilibrium simulation was carried out with an NPT ensemble at 300 K and 1 bar for 20 ns. The production simulation was carried out with an NVT ensemble at 300 K for 20 ns. RDF analysis and simulation snapshot visualization were visualized by VMD software[6].

Density Functional Theory (DFT) Calculation Methods. All molecular structures were optimized with the TPSSh functional and def2tzvp basis set, followed by HOMO and LUMO energy calculations using Gaussian 09.[7],[8] The SMD solvation model of tetrahydrofuran (ε = 7.4257) was employed to describe the

solvent environment[9]. The electrostatic potential (ESP) of solvent molecules was analysed in the Multiwfn program[10, 11].

Adsorption Energy Calculation Methods. First-principles calculations were performed using Vienna Ab-initio Simulation Package (VASP) version 5.4.1 with the Perdew-Burke-Ernzerhof (PBE) functional.[12] The (001) surface of hexagonal close-packed (hcp) Mg was modeled using a four-layer slab. The plane-wave cut-off energy was set to 500 eV, and the convergence criteria were 10^{-4} eV and 0.05 eV $Å^{-1}$ for electron energy and ion force. The adsorption energy (E_{ads}) was defined by the equation:

$$E_{ads} = E_{001\text{-solvents}} - E_{001} - E_{solvents}$$

where $E_{001\text{-solvents}}$ is the total energy of the Mg (001) surface with an adsorbed solvent molecule, $E_{surface}$ is the energy of the clean Mg (001) plane, and $E_{solvents}$ is the energy of the isolated solvent molecule, respectively. Therefore, a more negative E_{ads} value indicates a more exothermic adsorption process and thus a more favorable adsorption in all calculations.

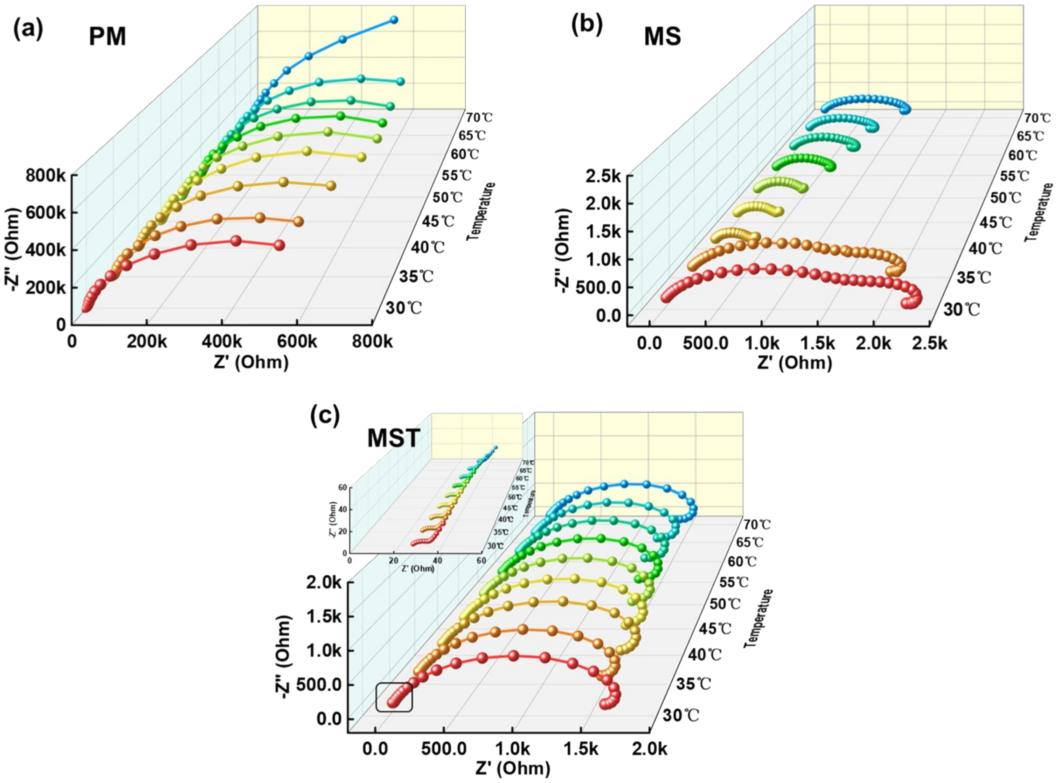

Fig.S1 EIS of the (a) pristine PM, (b) MS, and (c) MST electrolyte at different temperatures.

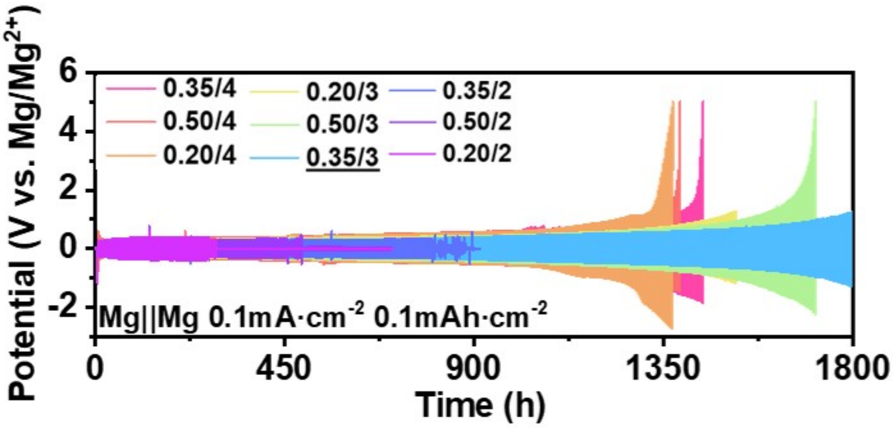

Fig.S2 Long-term cycling performance of Mg‖Mg symmetric cells at 0.1 mA cm^{-2} with a capacity of 0.1 mAh cm^{-2} in 0.5 M $Mg(TFSI)_2$ electrolytes containing different concentrations of $SiBr_4$ in mixed DME:TMSP solvents. In the legend, x/y denotes an electrolyte with x M $SiBr_4$ and a DME:TMSP volume ratio of 16:4, 17:3, or 18:2, corresponding to y = 4, 3, or 2, respectively.

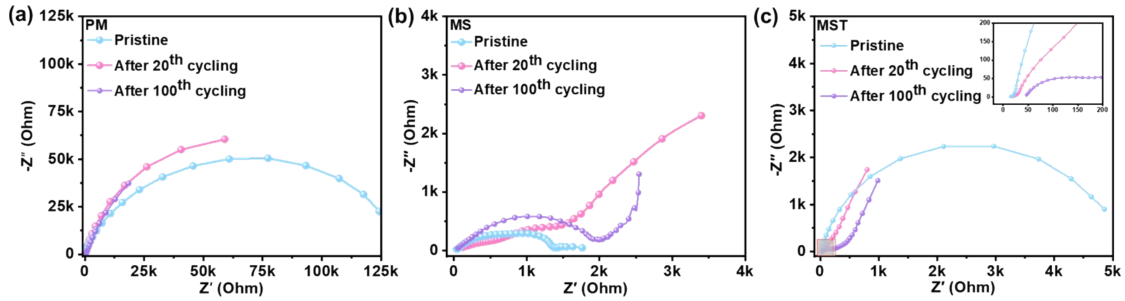

Fig.S3 EIS spectra of the Mg‖Mg symmetric cell using (a) the pristine PM, (b) MS, and (c) MST electrolyte at the initial state, after 20 cycles, and after 100 cycles.

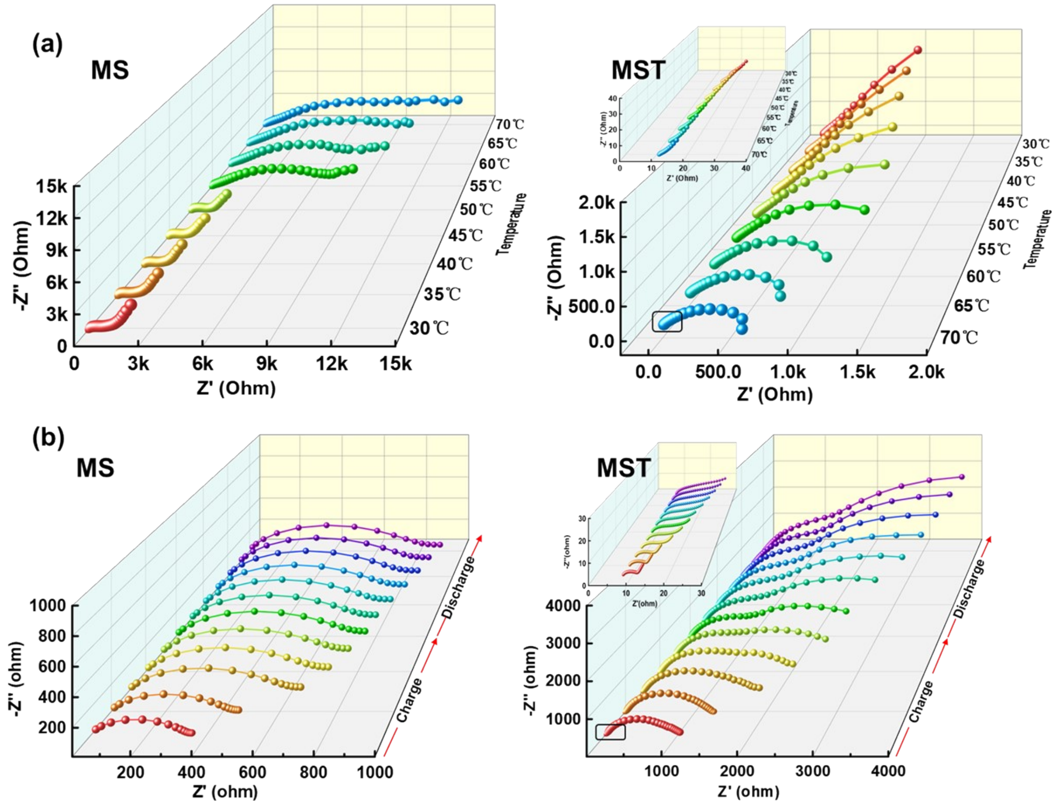

Fig.S4 (a) EIS spectra of Mg‖Mg symmetric cells with MS and MST electrolytes at different temperatures after 20 cycles. (b) In-situ EIS spectra were recorded every 20 min during cycling.

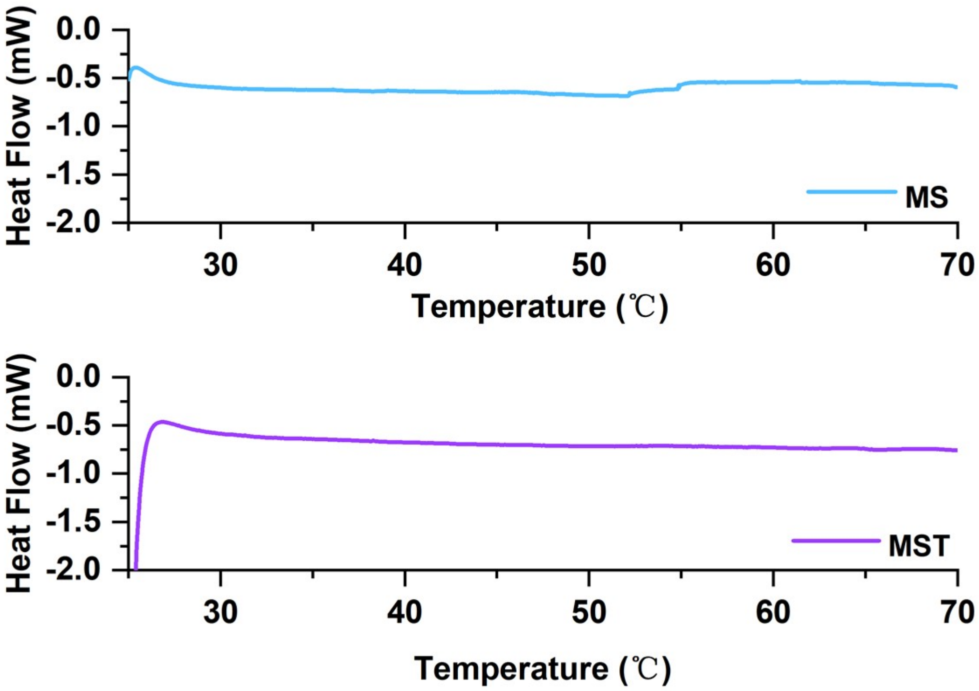

Fig.S5 Differential scanning calorimetry (DSC) curves of MS and MST electrolytes.

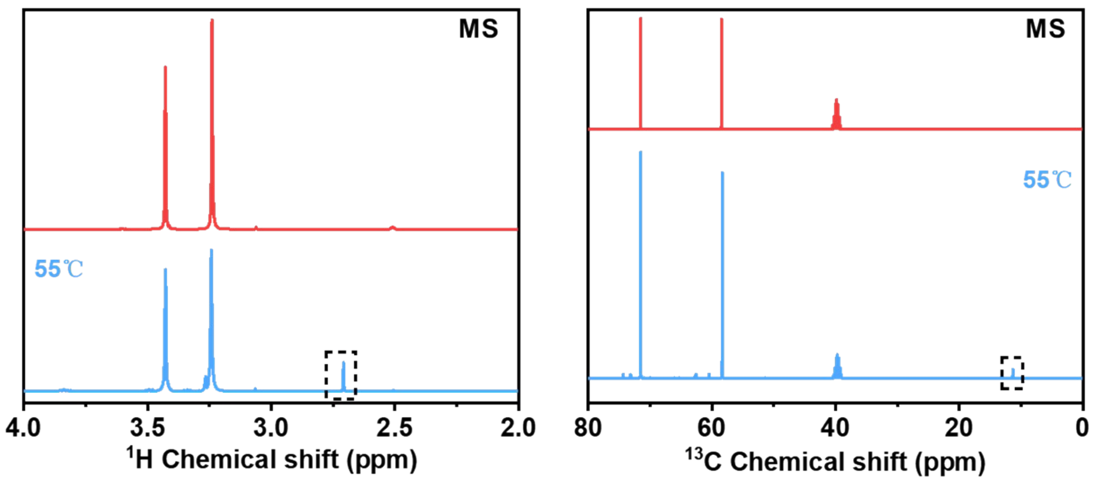

Fig.S6 Comparison of ^{1}H and ^{13}C NMR spectra of MS electrolyte before and after storage at 55 °C for 3h.

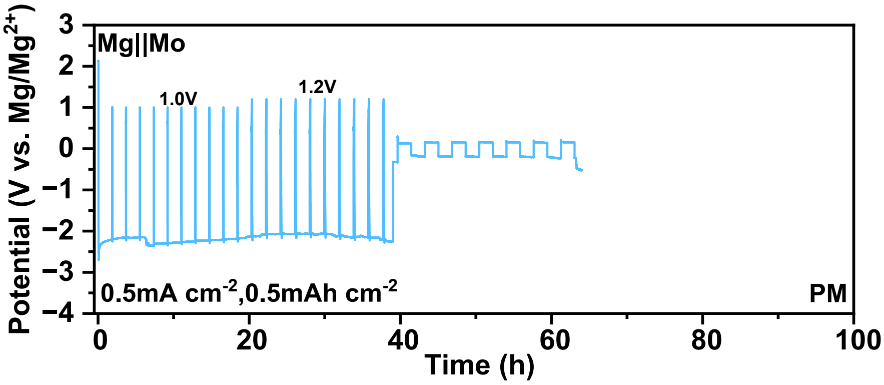

Fig.S7 Plating/stripping curves of the Mg‖Mo asymmetric cell using the pristine PM electrolyte at cut-off voltages ranging from 1.0 to 1.2 V.

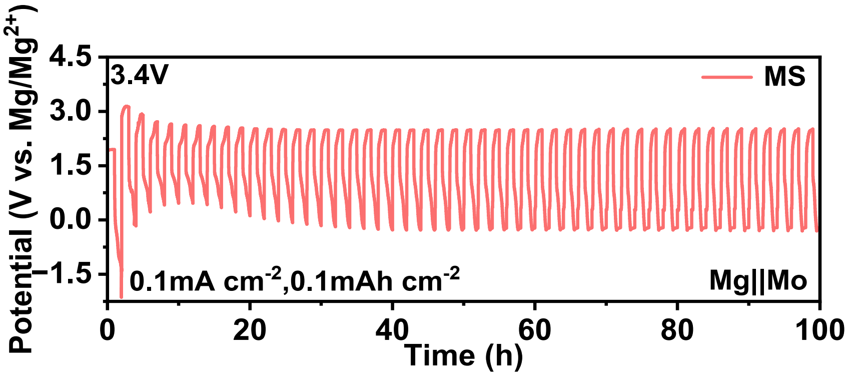

Fig.S8 Cycling performance of the Mg‖Mo asymmetric cell using MS electrolyte at a current density of 0.1 mA cm^{-2}, with a cut-off voltage of 3.4 V.

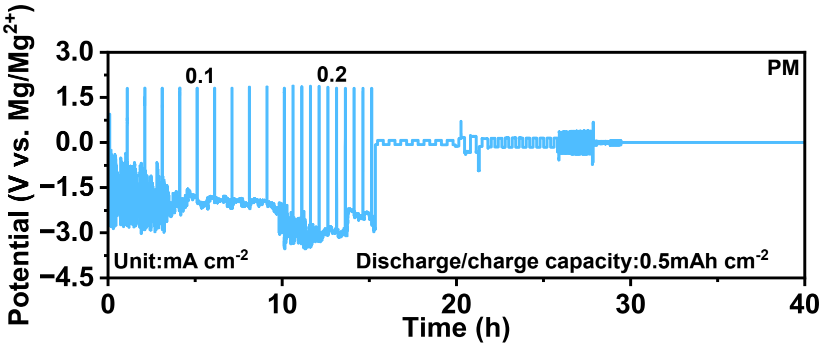

Fig.S9 Plating/stripping curves of the Mg‖Mo asymmetric cell with the pristine PM electrolyte at different densities.

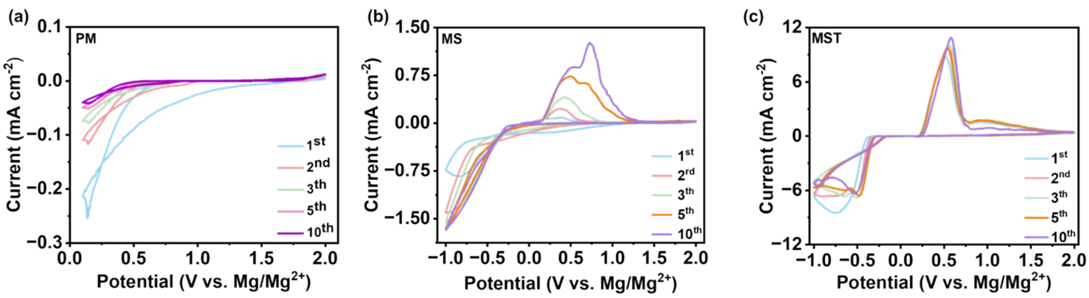

Fig.S10 CV curve of the Mg‖Mo asymmetric cell using the pristine (a) PM, (b) MS, and (c) MST electrolyte at a scanning rate of 0.5 mV s^{-1}.

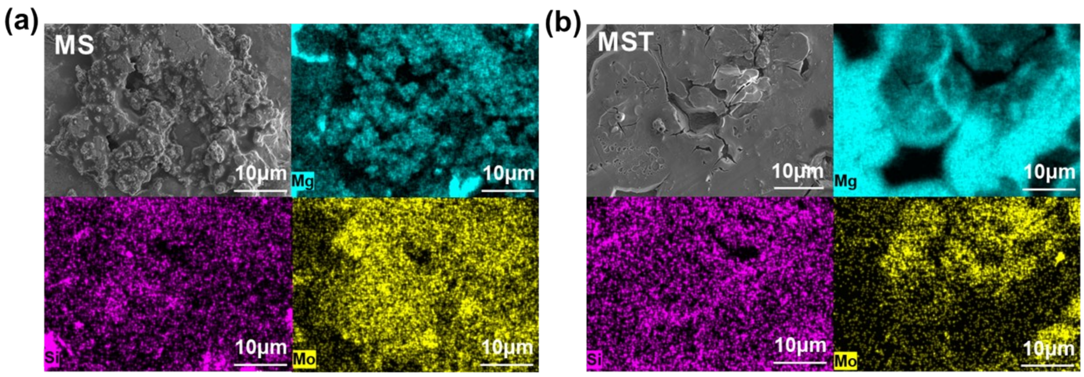

Fig.S11 SEM images of Mg deposited on Mo current collectors for 50 hours using (a) MS and (b) MST electrolyte, respectively, along with the corresponding EDS mapping images.

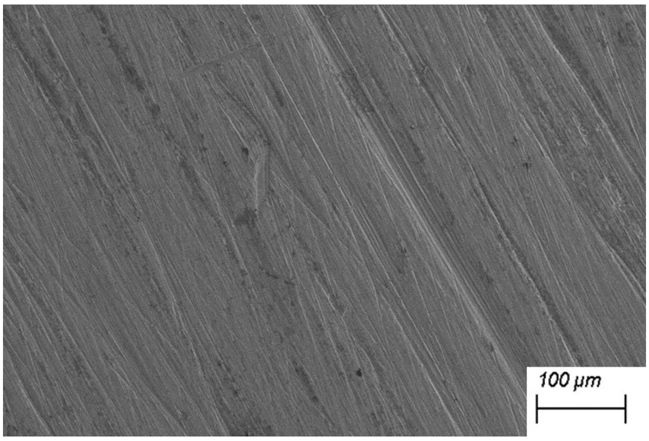

Fig.S12 SEM images of uncycled Mg anode.

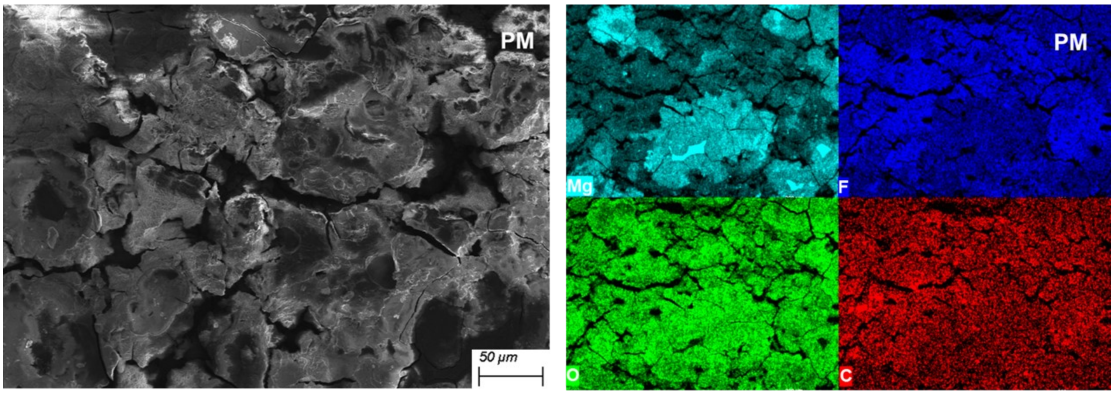

Fig.S13 SEM image of the Mg anode after cycling in the pristine PM electrolyte until short circuit, together with the corresponding EDS mapping.

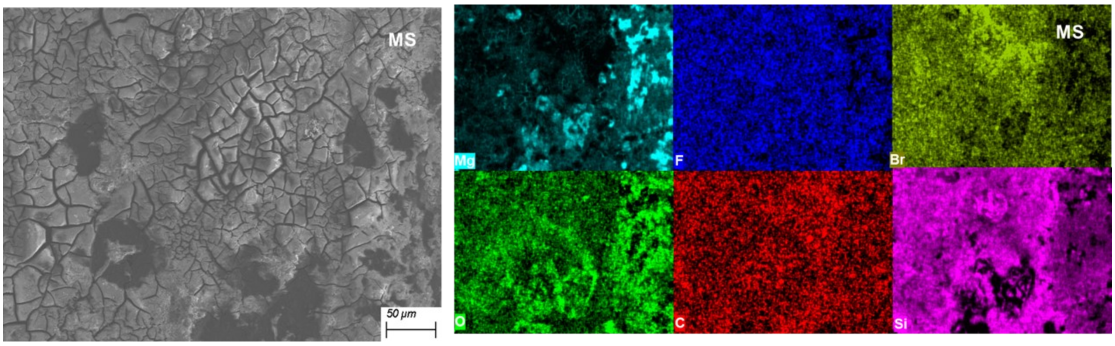

Fig.S14 SEM image of the Mg anode after 300 cycles in the MS electrolyte, with the corresponding EDS mapping.

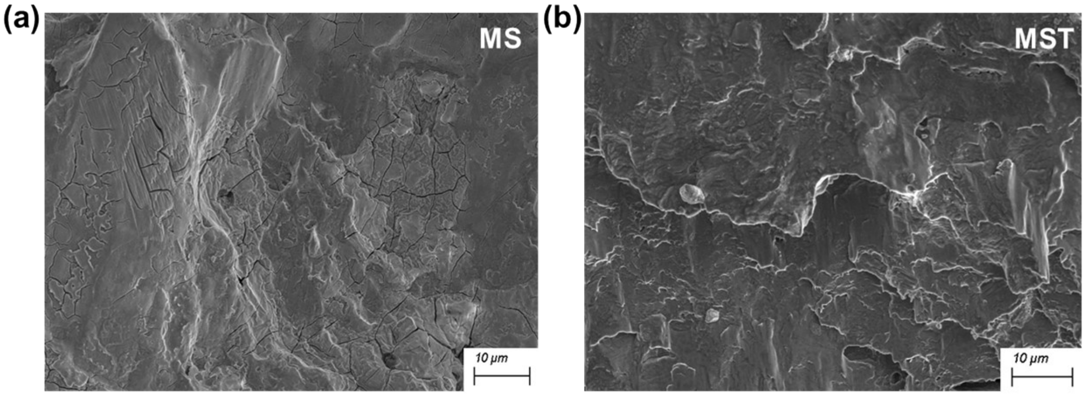

Fig.S15 Magnified SEM cross-sectional images of the SEI on Mg anodes after 100 cycles in (a) MS and (b) MST electrolytes.

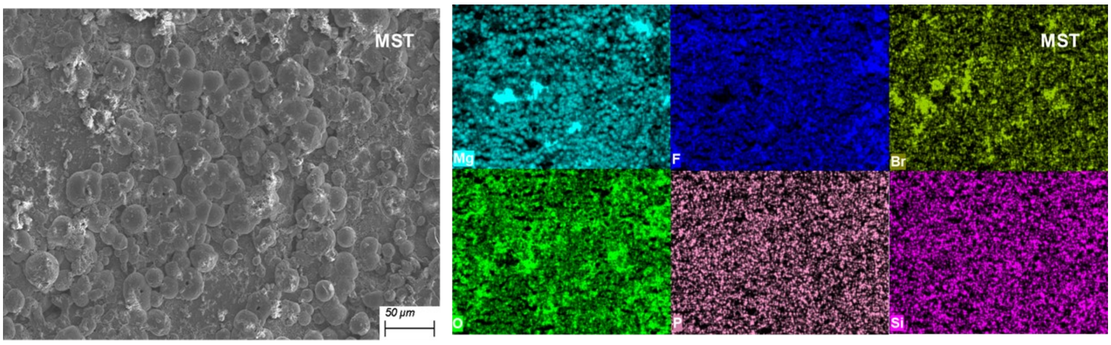

Fig.S16 SEM image of the Mg anode after 300 cycles in the MST electrolyte, with the corresponding EDS mapping.

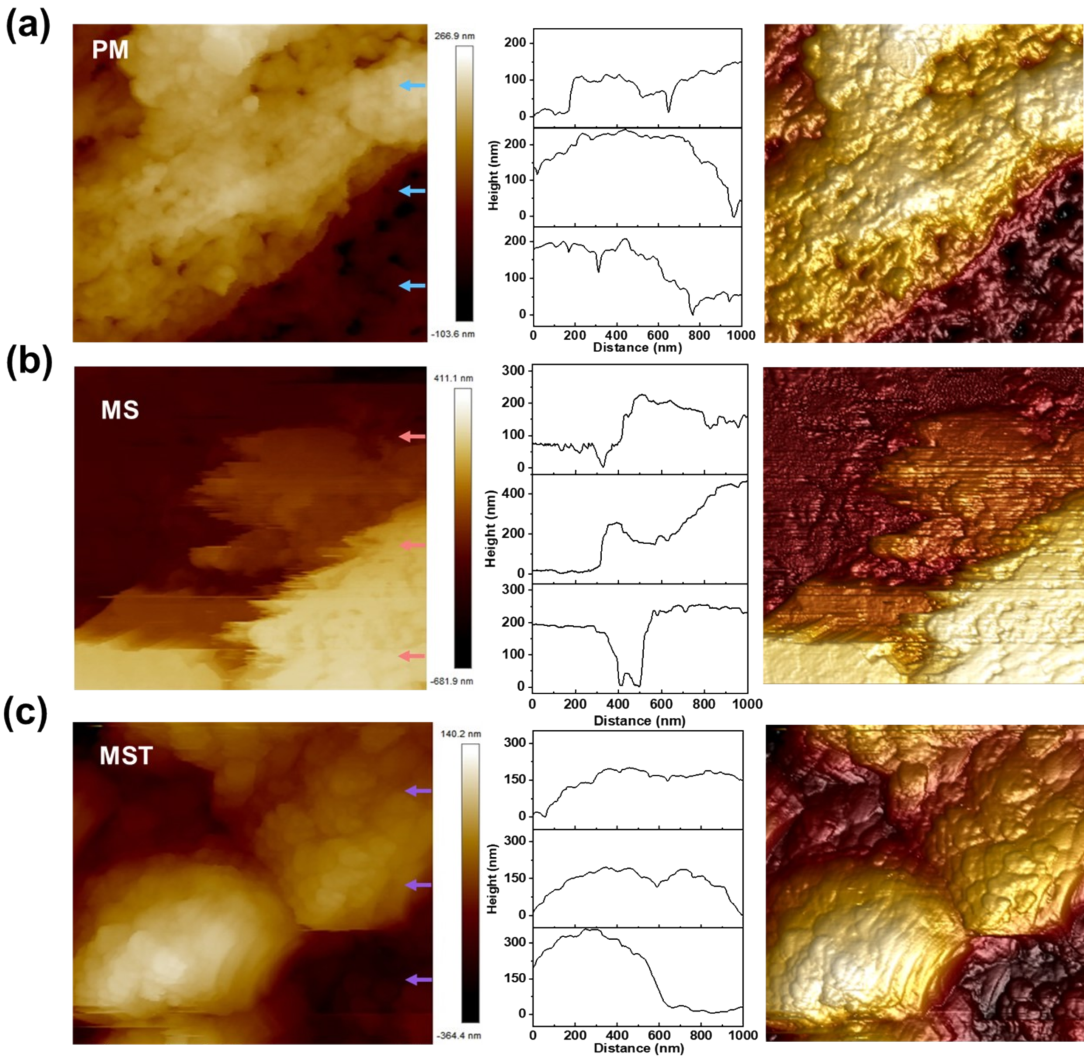

Fig.S17 The atomic force microscope (AFM) images of the Mg anode after 100 cycles in the (a) pristine PM, (b) MS, and (c) MST electrolyte, respectively.

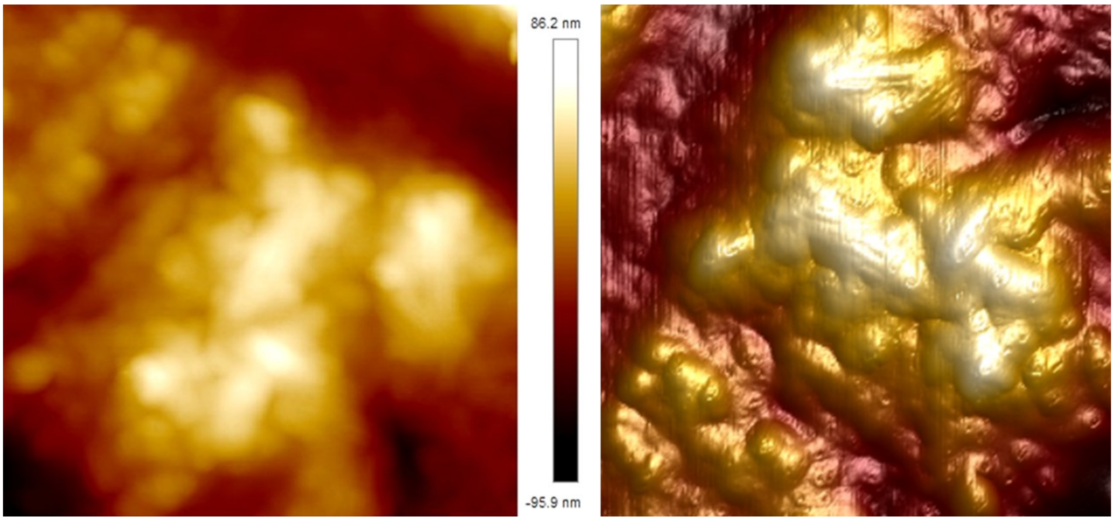

Fig.S18 AFM images of the Mg anode (fringe field) after 100 cycles in the MS electrolyte.

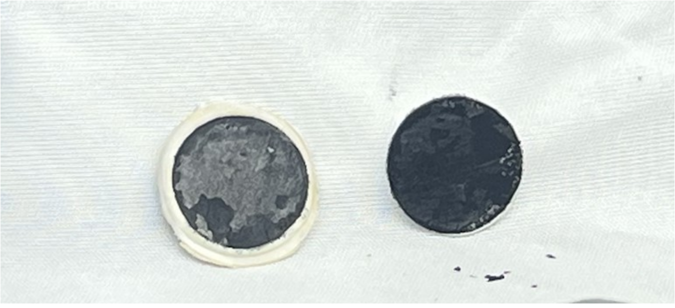

Fig.S19 Image of the Mg anode and separator after 100 cycles in the PM electrolyte.

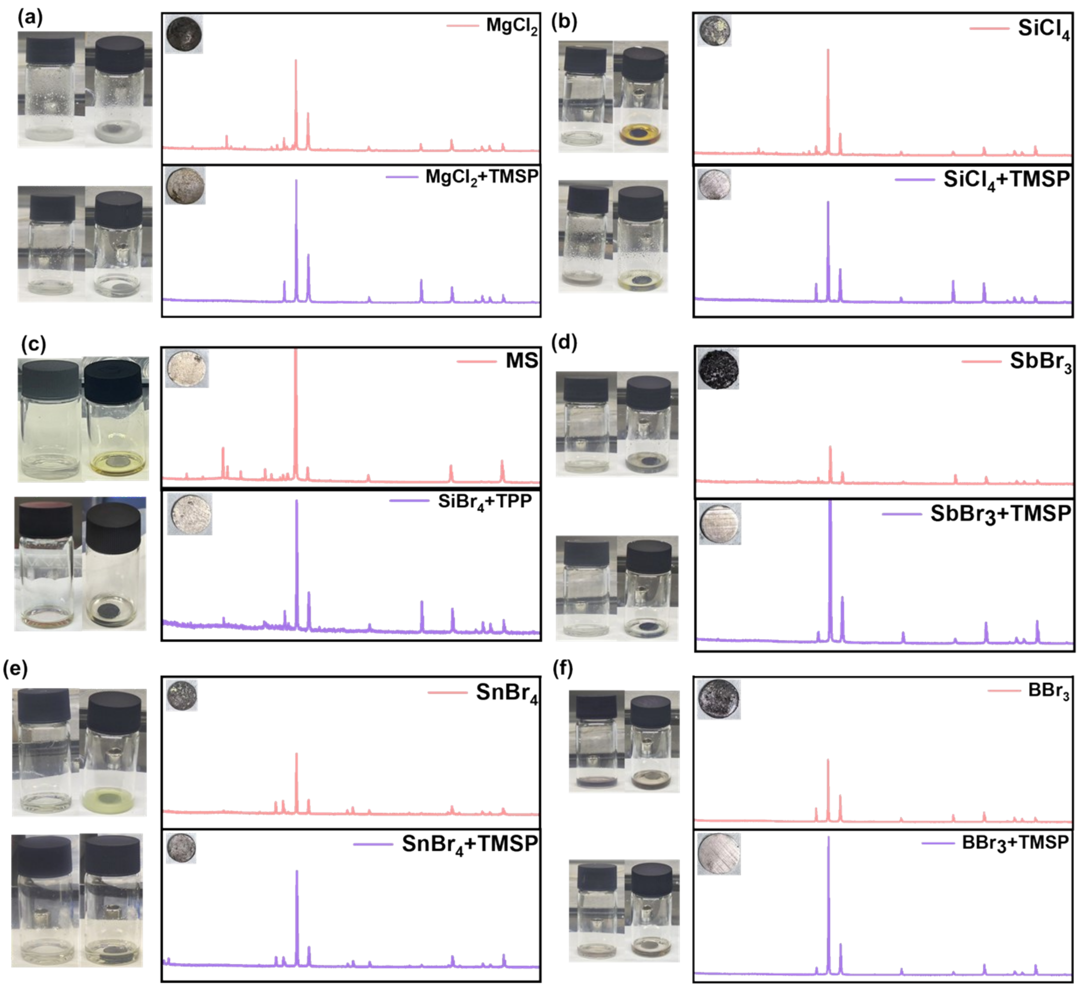

Fig.S20 Photographs of electrolytes containing individual halides ($MgCl_2$, $SiCl_4$, $SiBr_4$, $SbBr_3$, $SnBr_4$, BBr_3) or combined halide and phosphate ester components (e.g. TPP and TMSP), together with images of Mg foils after 0.5 h immersion and the corresponding XRD patterns.

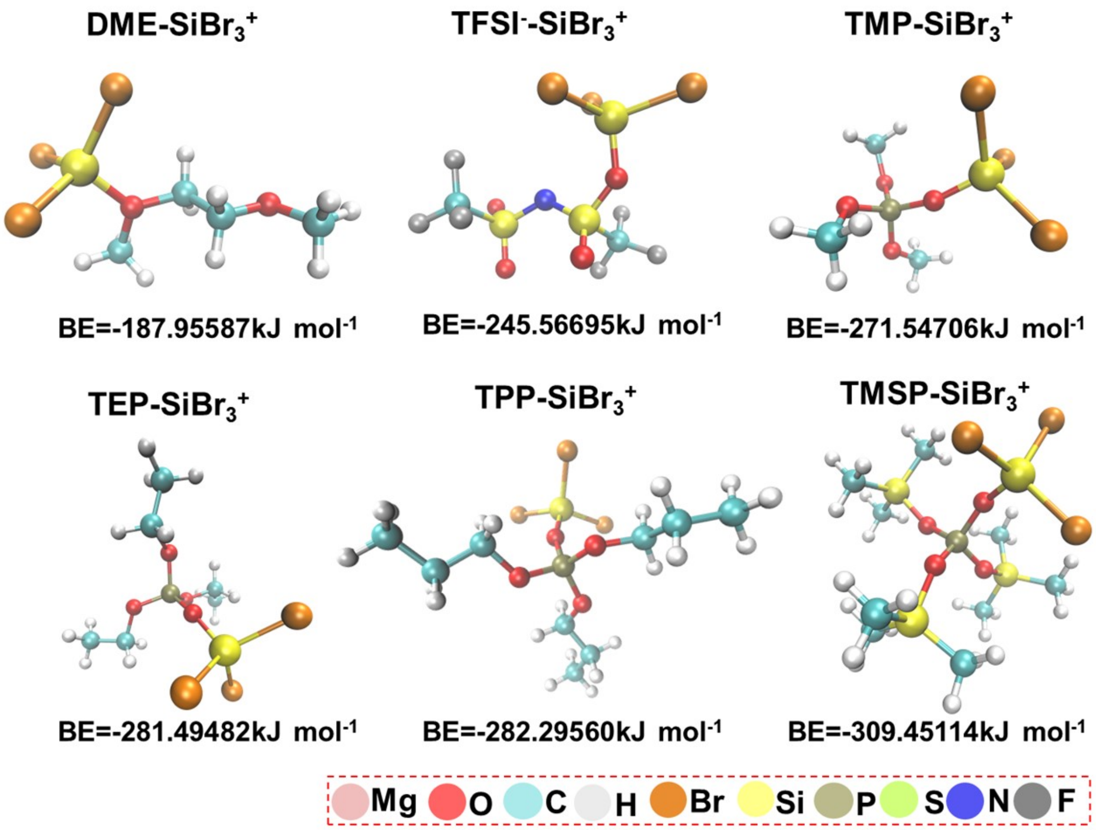

Fig.S21 The DFT calculation results of DME, $TFSI^-$, trimethyl phosphate (TMP), triethyl phosphate (TEP), TPP, TMSP, and the $SiBr_3^+$ cation.

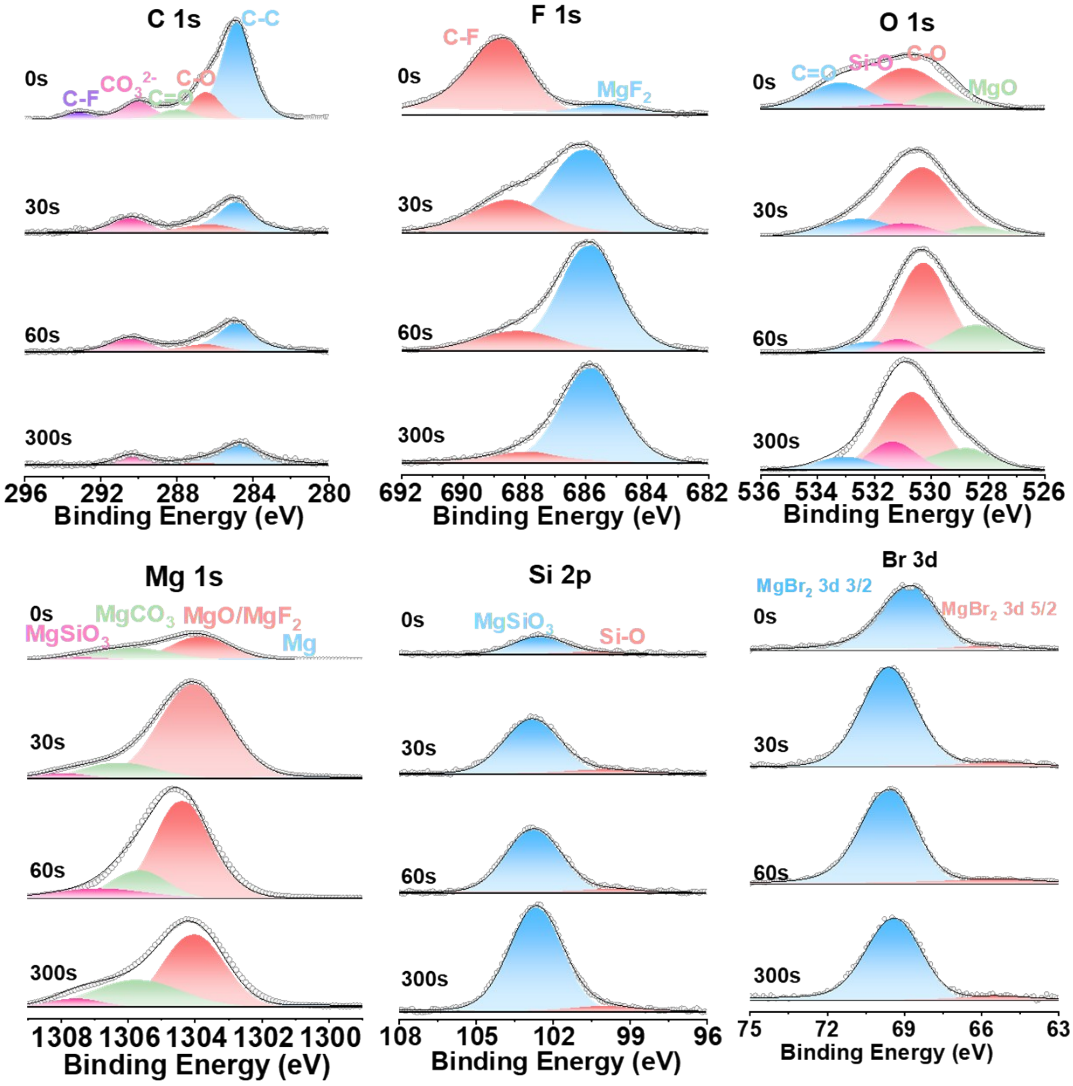

Fig.S22 XPS spectra of C 1s, F 1s, O 1s, Mg 1s, Si 2p, and Br 3d for Mg anode after 100 cycles in the MS electrolyte, measured at different Ar^{+} sputtering times.

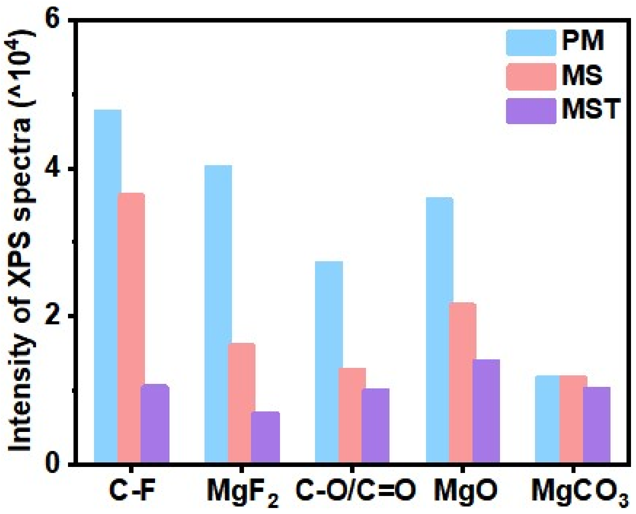

Fig.S23 The peak intensity of C-F, MgF_2, C-O/C=O, MgO, and $MgCO_3$ on the cycled Mg anode surface in pristine PM, MS, and MST electrolyte.

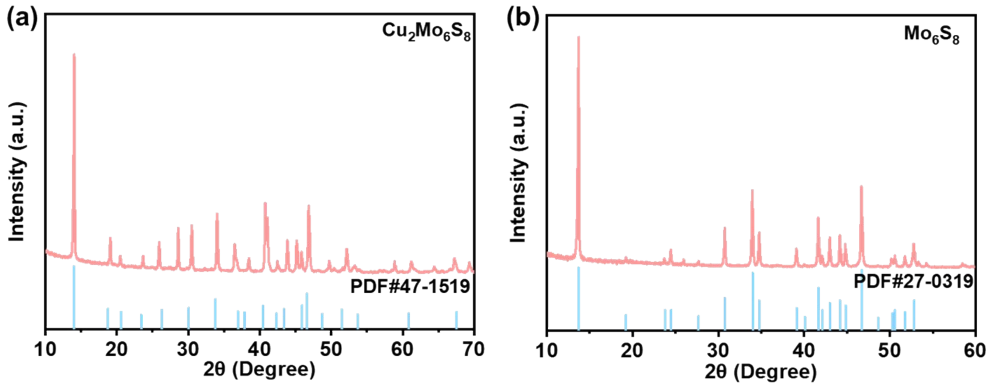

Fig.S24 XRD patterns of (a) intermediate products and (b) the final products during the cathode synthesis of the Chevrel Mo_6S_8 phase.

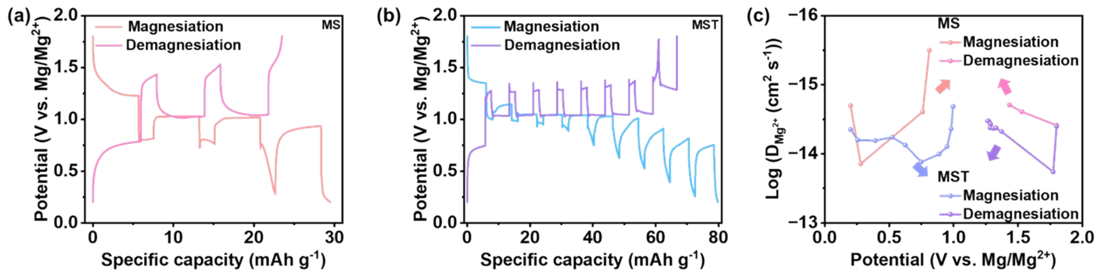

Fig.S25 GITT curve of the Mg‖Mo_6S_8 battery using the (a) MS and (b) MST electrolyte measured for 10 minutes at repeated constant current pulses of 0.5 C, followed by a relaxation of 30 minutes. (c) Diffusion coefficients calculated from the GITT curves using the MS electrolyte and MST electrolyte.

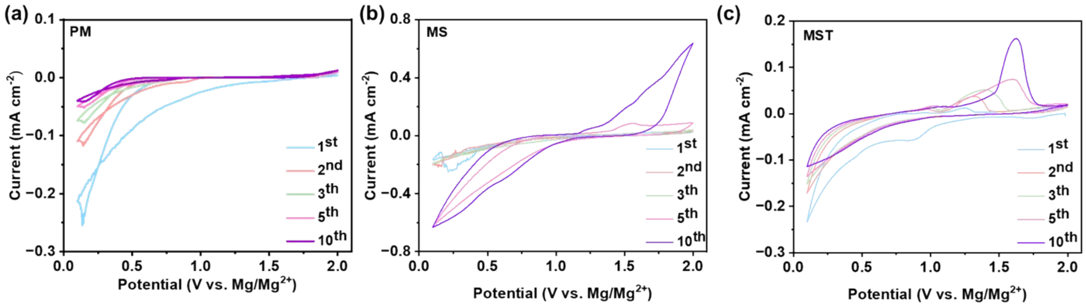

Fig.S26 CV curves of the Mg‖Mo_6S_8 full cell using the (a) pristine PM, (b) MS, and (c) MST electrolyte, respectively.

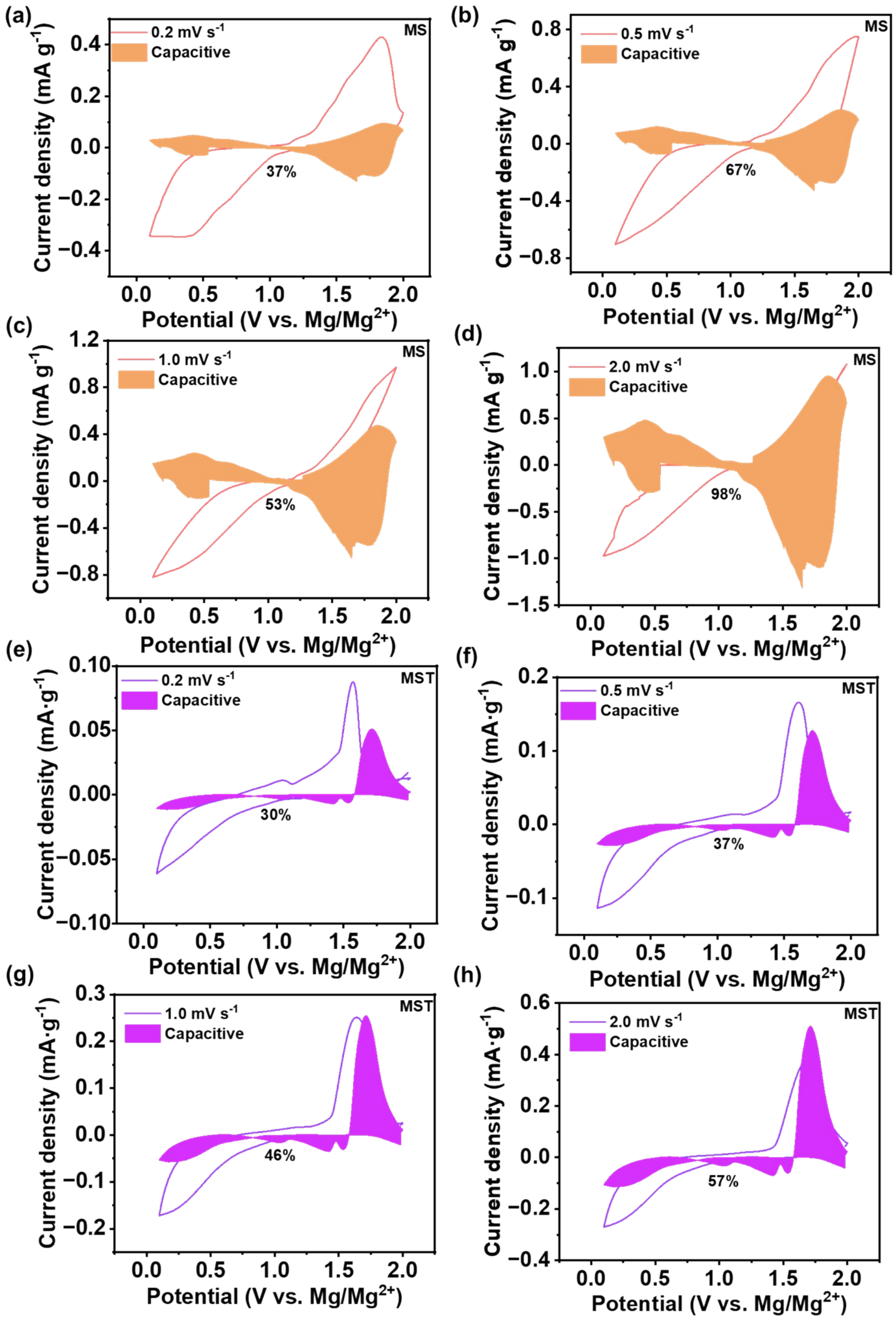

Fig.S27 Contribution ratio of capacitive and diffusion-controlled processes for Mg storage in Mo_6S_8 cathode with (a-d) MS and (e-h) MST electrolytes at various scan rates. The shaded pink regions represent the capacitive contribution, with the corresponding percentages marked.

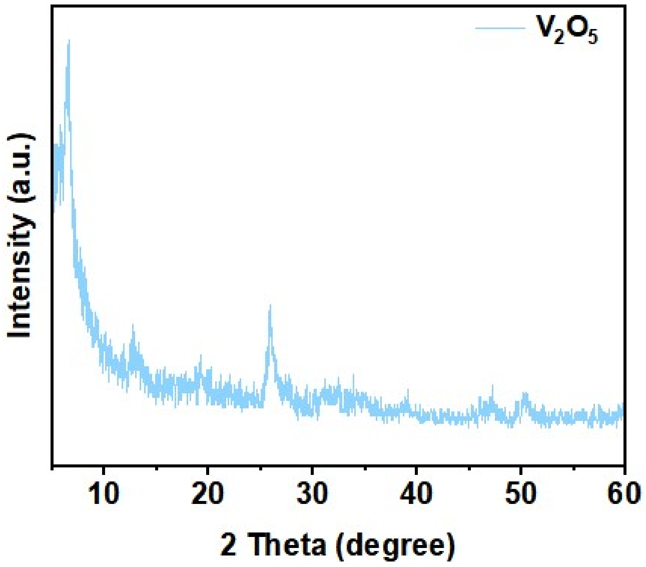

Fig.S28 XRD patterns of the polyaniline-intercalated-V_2O_5 cathode.

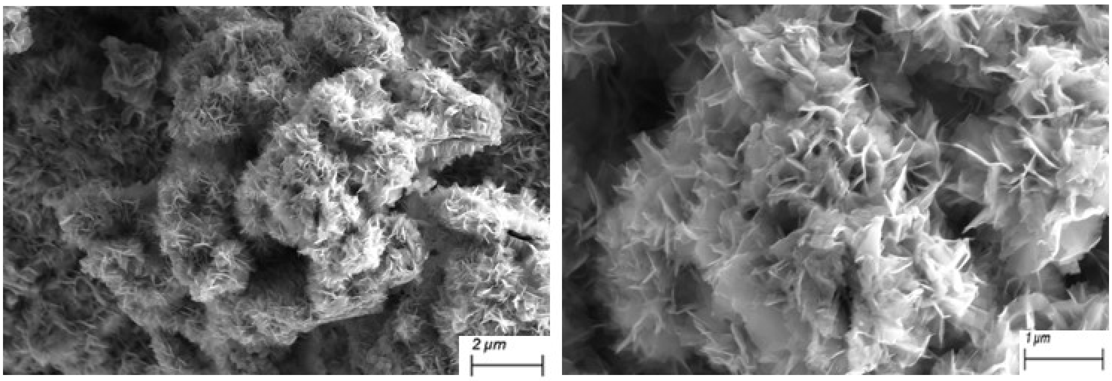

Fig.S29 SEM image of the polyaniline-intercalated V_2O_5 powder.

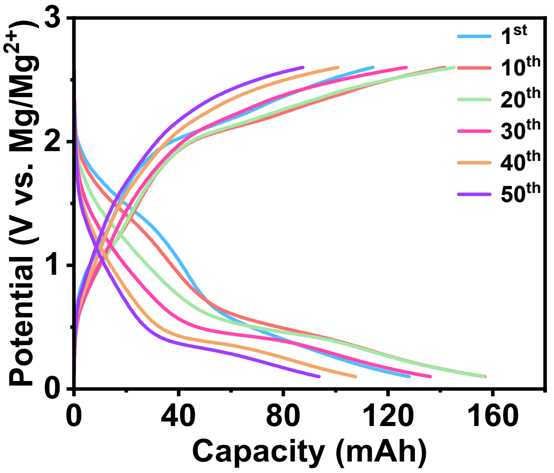

Fig.S30 Selected charge-discharge profiles of Mg‖V_2O_5 cells with the MST electrolyte at 0.5 C.

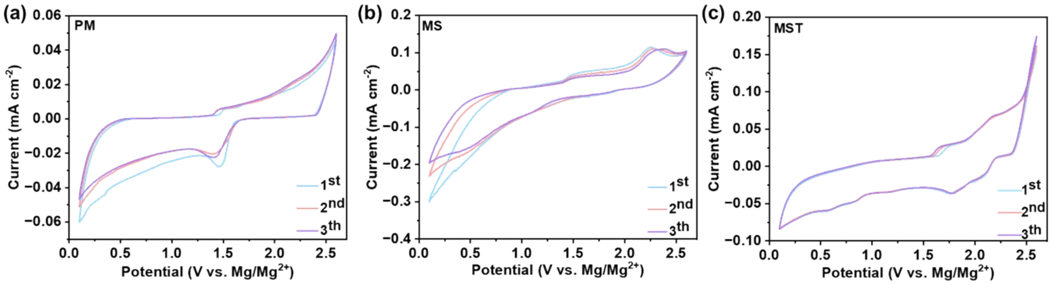

Fig.S31 CV curves of Mg‖V_2O_5 full cells with the (a) pristine PM, (b) MS, and (c) MST electrolyte, respectively.

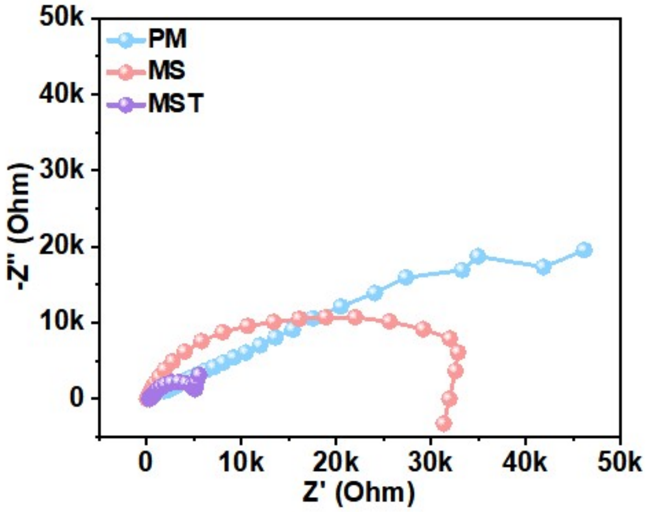

Fig.S32 EIS spectra of Mg‖V_2O_5 full cells before cycling in the pristine PM, MS, and MST electrolytes.

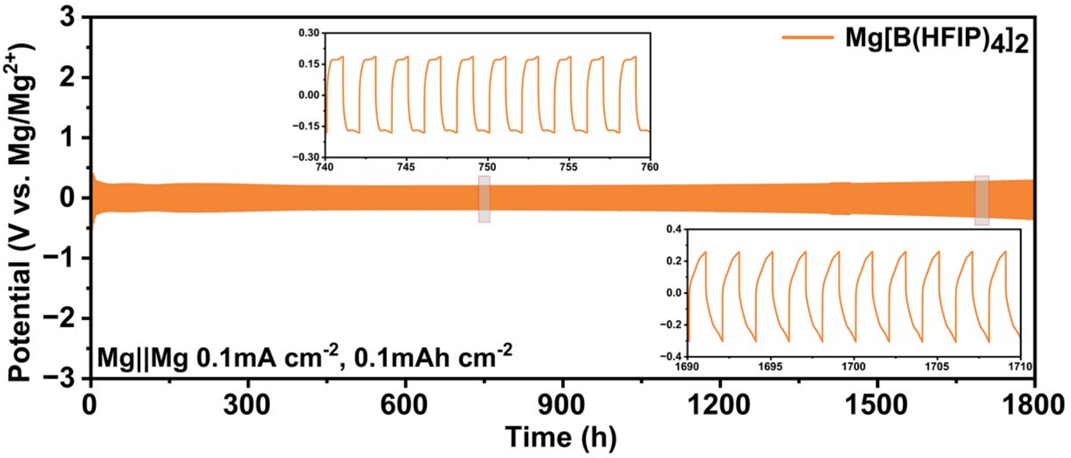

Fig.S33. Cyclic performance of Mg‖Mg symmetric batteries assembled by 0.3 M $Mg(B(HFIP)_4)_2$ + 0.35 M $SiBr_4$ in DME: TMSP (17:3, v/v).

Table S1. A systematic comparison with previously reported $Mg(TFSI)_2/Mg(OTf)_2$-based electrolyte systems.

Electrolyte compositions	Plating potential	Cycle lifespan	Coulombic efficiency	Electrochemical Window	Cathodes	Capacity	Capacity retention ratio	Cycle lifespan (cycles)	Refs
0.5 M $Mg(TFSI)_2$ + 2.0 M DMA in THF	−0.45 V	26 h	56.0 %						[13]
0.25 M $Mg(TFSI)_2$ + 2.0 M DMA in THF	−0.2 V	23 cycles	98.0 %						[14]
0.5 M $Mg(TFSI)_2$ in (DME+MOPA-1)	−0.1 V	190 h	99.5 %	3.80 V	$Mg_{0.15}MnO_2$	93.0	≈74.5 %	200	[15]
0.25 M $Mg(OTf\ )_2$ in (G2+MOEA)	−0.26 V	5000 h	98.0 %	2.80 V	Mo_6S_8/Cu_3Se_2	59.3/67.0	≈88 % /≈42 %	1000/200	[16]
0.25 M $Mg(OTf\ )_2$ in (G2+DME+MOEA)	−0.15 V	1000 h	98.0 %	3.50 V	Mo_6S_8	52.5	84 %	300	[17]
0.5 M $Mg(TFSI)_2$ in (DME+aniline)	−0.15 V	150 h	70.0 %						[18]
0.1 M $Mg(TFSI)_2$ in MOPA-2	−0.05 V	350 h	95.9 %						[19]
0.25 M $Mg(TFSI)_2$ in (G2+DMAPA)	−0.1 V	320 h	92.0 %						[20]
0.5 M $Mg(TFSI)_2$ in (DME+TMP)	−0.2 V	300 h	79.0 %						[21]
0.4 M $Mg(TFSI)_2$ in (G2+IBA)	−0.16 V	100 h	90.6 %	4.03 V	Mo_6S_8	30.0	≈18.6 %	600	[22]
0.25 M $Mg(TFSI)_2$ in (G4+PDA)					CO_2	600.0		70	[23]
0.18 M $Mg(TFSI)_2$ + 0.1 M LiOTf in (DME+TMP)	−0.22 V	450 h	98.0 %	3.50 V	Mo_6S_8	53.0	≈82.8%	200	[5]
0.4 M $Mg(TFSI)_2$ in (DME+G2+BTFE+TEP)	−0.15 V	7000 h	95.2 %	4.16 V	PANI	113.0	72 %	400	[24]
0.125 M $Mg(OTf\ )_2$ + 0.125 M $Mg(HMDS)_2$ + 0.5 M TBAOTF in (DME+G3)	−0.29 V	550 h	90.0 %						[25]
0.5 M $Mg(TFSI)_2$ + 2% HpMS in G3	−0.4 V	160 cycles	66.2 %						[26]
0.5 M $Mg(TFSI)_2$ + 0.15 M $Mg[B(OPh)_3H]_2$ in G2	−0.23 V	20 h	74.0 %						[27]
0.5 M $Mg(TFSI)_2$+0.3M $SbBr_3$ in DME	-0.25 V	3100 h		2.90 V	Mo_6S_8/CuS	36.7/85.2	71.35 % /82.8 %	500/300	[28]
0.25 M $Mg(TFSI)_2$+0.4M BrFB in G2	-0.19 V	7000 h	98.15 %	2.81 V	CuS		≈55.4 %	65	[29]
**0.5M $Mg(TFSI)_2$+0.35M $SiBr_4$ in DME**	**−0.42 V**	**1800 h**	**93.52 %**	**3.76V**	**Mo_6S_8**	**22.5**	**39.35 %**	**500**	**This Work**
**0.5M $Mg(TFSI)_2$+0.35M $SiBr_4$ in (DME+TMSP)**	**−0.14 V**	**1800 h**	**99.97 %**	**3.94 V**	**Mo_6S_8/V_2O_5**	**45.3/93.6**	**56.63 % /73.13 %**	**500/50**	**This Work**

DMA: Dimethylamine

THF: Tetrahydrofuran

DME: Dimethoxyethane

MOPA-1: 1-.methoxy-2-prpylamine

G2: Diethylene Glycol Dimethyl Ether

MOEA: 2-Methoxyethylamine

MOPA-2: 3-methoxypropylamine

DMAPA: 3-dimethylaminopropylamine

TMP: Trimethyl phosphate

IBA: Isobutylamine

G4: Tetraethylene glycol dimethyl ether

PDA: 1,3-propyleneamine

BTFE: Bis(2,2,2-trifluoroethyl) ether

TEP: Triethyl phosphate

G3: Triethylene glycol dimethyl ether

TBAOTF: Tetrabutylammonium Triflate

HpMS: Heptamethyldisilazane

BrFB: 3-Bromofluorobenzene

TMSP: Tris(trimethylsilyl) phosphate

References

1. E. Lancry, E. Levi, A. Mitelman, S. Malovany and D. Aurbach, *Journal of Solid State Chemistry*, 2006, **179**, 1879-1882.

2. S. Liu, H. Zhu, B. Zhang, G. Li, H. Zhu, Y. Ren, H. Geng, Y. Yang, Q. Liu and C. C. Li, *Advanced Materials*, 2020, **32**, 202001113.
3. M. J. Abraham, T. Murtola, R. Schulz, S. Páll, J. C. Smith, B. Hess and E. Lindahl, *SoftwareX*, 2015, **1-2**, 19-25.
4. L. Martínez, R. Andrade, E. G. Birgin and J. M. Martínez, *Journal of Computational Chemistry*, 2009, **30**, 2157-2164.
5. S. Wang, K. Wang, Y. Zhang, Y. Jie, X. Li, Y. Pan, X. Gao, Q. Nian, R. Cao, Q. Li, S. Jiao and D. Xu, *Angewandte Chemie International Edition*, 2023, **62**, e202304411.
6. A. D. William Humphrey, and Klaus Schulten, *J. Mol. Graph.*, 1996, 33-38.
7. B. Wu, Q. Liu, D. Mu, H. Xu, L. Wang, L. Shi, L. Gai and F. Wu, *RSC Advances*, 2016, **6**, 51738-51746.
8. P. J. S. F.J. Devlin, J.R. Cheeseman, M.J. Frisch, *J. Phys. Chem. A*, 1997, 6322-6333.
9. C. J. C. A.V. Marenich, D.G. Truhlar, *J. Phys. Chem. B*, 2009, 6378–6396.
10. T. Lu and F. Chen, *Journal of Computational Chemistry*, 2011, **33**, 580-592.
11. T. Lu, *The Journal of Chemical Physics*, 2024, **161**, 082503.
12. J. Hafner, *Journal of Computational Chemistry*, 2008, **29**, 2044-2078.
13. S. Fan, G. M. Asselin, B. Pan, H. Wang, Y. Ren, J. T. Vaughey and N. Sa, *ACS Applied Materials & Interfaces*, 2020, **12**, 10252-10260.
14. S. Fan, S. Cora and N. Sa, *ACS Applied Materials & Interfaces*, 2022, **14**, 46635-46645.
15. X. J. Singyuk Hou, Karen Gaskell, Peng-fei Wang, Luning Wang, Jijian Xu, Ruimin Sun, Oleg Borodin, and Chunsheng Wang, *Science*, 2021, DOI: 10.1126/science.abg3954, 172 - 178.
16. D. Zhang, Y. Wang, Y. Yang, Y. Zhang, Y. Zhao, M. Pan, Y. Sun, S. Chen, X. Liu, J. Wang and Y. NuLi, *Advanced Energy Materials*, 2023, **13**, 2301795.
17. Y. Du, Y. Chen, S. Tan, J. Chen, X. Huang, L. Cui, J. Long, Z. Wang, X. Yao, B. Shang, G. Huang, X. Zhou, L. Li, J. Wang and F. Pan, *Energy Storage Materials*, 2023, **62**.
18. Y. Liu, W. Zhao, Z. Pan, Z. Fan, M. Zhang, X. Zhao, J. Chen and X. Yang, *Angewandte Chemie International Edition*, 2023, **62**, 102939.
19. F. Wang, H. Hua, D. Wu, J. Li, Y. Xu, X. Nie, Y. Zhuang, J. Zeng and J. Zhao, *ACS Energy Letters*, 2022, **8**, 780-789.

20. M. Wang, W. Sun, K. Zhang, Z. Zhang, A. Du, S. Dong, J. Zhang, J. Liu, X. Chen, Z. Zhou, F. Li, Z. Li, G. Li and G. Cui, *Energy & Environmental Science*, 2024, **17**, 630-641.
21. W. Zhao, Z. Pan, Y. Zhang, Y. Liu, H. Dou, Y. Shi, Z. Zuo, B. Zhang, J. Chen, X. Zhao and X. Yang, *Angewandte Chemie International Edition*, 2022, **61**, e202205187.
22. J. Zhang, N. Yuan, J. Liu, X. Guo, X. Chen, Z. Zhou, Z. Zhang and G. Li, *Journal of Energy Chemistry*, 2024, **98**, 615-622.
23. C. Peng, L. Xue, Z. Zhao, L. Guo, C. Zhang, A. Wang, J. Mao, S. Dou and Z. Guo, *Angewandte Chemie International Edition*, 2023, **63**, e202313264.
24. C. Li, R. D. Guha, A. Shyamsunder, K. A. Persson and L. F. Nazar, *Energy & Environmental Science*, 2024, **17**, 190-201.
25. D. Chinnadurai, Y. Li, C. Zhang, G. Yang, W. Y. Lieu, S. Kumar, Z. Xing, W. Liu and Z. W. Seh, *Nano Letters*, 2023, **23**, 11233-11242.
26. S.-J. Kang, H. Kim, S. Hwang, M. Jo, M. Jang, C. Park, S.-T. Hong and H. Lee, *ACS Applied Materials & Interfaces*, 2018, **11**, 517-524.
27. S. Hebié, H. P. K. Ngo, J.-C. Leprêtre, C. Iojoiu, L. Cointeaux, R. Berthelot and F. Alloin, *ACS Applied Materials & Interfaces*, 2017, **9**, 28377-28385.
28. C. Wu, L. Xue, A. Wang, L. Guo, W. Tang, S. X. Dou and C. Peng, *Journal of Power Sources*, 2024, **605**, 42234517.
29. G. Li, L. Cao, M. Lei, K. Chen and C. Li, *Energy & Environmental Science*, 2025, **18**, 7254-7266.
